# In vivo photoreceptor base editing ameliorates rhodopsin-E150K autosomal-recessive retinitis pigmentosa in mice

**DOI:** 10.1073/pnas.2416827121

**Published:** 2024-11-18

**Authors:** Samuel W. Du, Gregory A. Newby, David Salom, Fangyuan Gao, Carolline Rodrigues Menezes, Susie Suh, Elliot H. Choi, Paul Z. Chen, David R. Liu, Krzysztof Palczewski

**Affiliations:** ^a^Gavin Herbert Eye Institute—Center for Translational Vision Research, Department of Ophthalmology, University of California, Irvine, CA 92617; ^b^Department of Physiology and Biophysics, University of California, Irvine, CA 92617; ^c^Merkin Institute of Transformative Technologies in Healthcare, Broad Institute of Massachusetts Institute of Technology and Harvard, Cambridge, MA 02142; ^d^Department of Chemistry and Chemical Biology, Harvard University, Cambridge, MA 02138; ^e^HHMI, Harvard University, Cambridge, MA 02138; ^f^Department of Genetic Medicine, Johns Hopkins University, Baltimore, MD 21205; ^g^Department of Biomedical Engineering, Johns Hopkins University, Baltimore, MD 21205; ^h^Koch Institute for Integrative Cancer Research, Massachusetts Institute of Technology, Cambridge, MA 02139; ^i^Department of Chemical Engineering, Massachusetts Institute of Technology, Cambridge, MA 02139; ^j^Department of Chemistry, University of California, Irvine, CA 92697; ^k^Department of Molecular Biology and Biochemistry, University of California, Irvine, CA 92697

**Keywords:** rhodopsin, base editing, prime editing, retinitis pigmentosa

## Abstract

Rhodopsin, the prototypical class-A G-protein coupled receptor, is a highly sensitive receptor for light that enables phototransduction in rod photoreceptors. Rhodopsin plays not only a sensory role but also a structural role as a major component of the rod outer segment disc, comprising over 90% of the protein content of the disc membrane. Mutations in *RHO* which lead to structural or functional abnormalities, including the autosomal recessive E150K mutation, result in rod dysfunction and death. Therefore, correction of deleterious rhodopsin mutations could rescue inherited retinal degeneration, as demonstrated for other visual genes such as *RPE65* and *PDE6B.* In this study, we describe a CRISPR/Cas9 adenine base editing strategy to correct the E150K mutation and demonstrate precise in vivo editing in a *Rho*-E150K mouse model of autosomal recessive retinitis pigmentosa (RP). Using ultraviolet-visible spectroscopy, mass spectrometry, and the G-protein activation assay, we characterized wild-type rhodopsin and rhodopsin variants containing bystander base edits. Subretinal injection of dual-adeno-associated viruses delivering our base editing strategy yielded up to 44% *Rho* correction in homozygous *Rho*-E150K mice. Injection at postnatal day 15, but not later time points, restored rhodopsin expression, partially rescued retinal function, and partially preserved retinal structure. These findings demonstrate that in vivo base editing can restore the function of mutated structural and functional proteins in animal models of disease, including rhodopsin-associated RP and suggest that the timing of gene-editing is a crucial determinant of successful treatment outcomes for degenerative genetic diseases.

## Significance

CRISPR/Cas9-based therapies could be beneficial for inherited retinal disorders (IRDs). IRDs are caused by mutations in genes expressed within the eye that have enzymatic, regulatory, or structural functions. Several major classes of IRDs are caused by mutations in rhodopsin, a light-sensitive molecule whose function includes both enzymatic and structural roles. Our study demonstrates precision genome-editing in an autosomal-recessive form of rhodopsin-associated retinitis pigmentosa. We characterized a base editing strategy and assessed editing and functional outcomes after delivery of split-intein dual-adeno-associated viruses encoding an adenine base editor to effect in vivo base editing of mouse rod photoreceptors and demonstrated restoration of wild-type rhodopsin production. Further development and delivery of base editors to the retina may expand treatment options for IRD patients.

Image-forming vision depends on inputs from the light-sensitive neurons called rod and cone photoreceptors, which sense dim and bright light, respectively (1). Photoreceptors, in turn, depend on light-sensitive proteins known as opsins located in their outer segments to capture and transduce photons of light. The rod opsin, known as rhodopsin, was discovered by Böll in 1876, subsequently determined to be a G-protein coupled receptor (GPCR), and the first GPCR crystallized and structurally solved (2–4). Composed of seven transmembrane domains, rhodopsin binds the chromophore 11-*cis*-retinal through a protonated Schiff-base linkage to the Lys^296^ residue (5); upon stimulation by a photon, the 11-*cis*-retinal photoisomerizes to all-*trans*-retinal, thus inducing a conformational change and propagating the signal (6).

Rhodopsin is not only a sensory protein but also plays a major structural role in the rod photoreceptor. Rods utilize a modified primary cilium, termed the rod outer segment (ROS), in which the phototransduction proteins are assembled (7, 8). Depending on the species, the ROS is composed of varying numbers of separate layered and regularly spaced membranous discs within its plasma membrane; for example, mouse ROS have around 800 discs, while amphibians have much larger rods with a corresponding increase in ROS disc number. Rhodopsin, at an approximate concentration of 3 to 5 mM, is the most abundant protein within the ROS disc, representing over 90% of the protein content and 50% of the ROS surface area (9). In mice, each disc contains approximately 8 × 10^4^ rhodopsin molecules, totaling ~4 × 10^14^ molecules per eye (~650 pmol/eye) (10, 11). Within the disc, rhodopsin has been shown to form dimers and higher-order multimers and is essential for overall ROS morphogenesis (12–14). Thus, *Rho*^−/−^ mice do not form the ROS and undergo rod degeneration, while *Rho*^+/−^ mice exhibit ROS which have ~60% of the volume of ROS in *Rho*^+/+^ mice (11). Rhodopsin is also rapidly turned over, as the entire ROS in each rod is phagocytosed and completely replaced roughly every 10 d in rodents (15, 16), and at a similar frequency in primates (17). Accordingly, sustained high expression of rhodopsin is critical for maintaining the structure and function of the ROS.

The first genetic mutation linked to retinitis pigmentosa (RP) was the P23H-rhodopsin mutation, causative for autosomal dominant RP (adRP) (18). This mutation is responsible for a substantial number of adRP cases, accounting for roughly 40% of *RHO*-associated adRP in the United States, and it is one of the best-characterized rhodopsin mutants (19, 20). Subsequently, over 150 adRP-causing mutations have been identified. Collectively, these mutant rhodopsins impact fundamental cellular processes such as Golgi trafficking, outer segment targeting, protein folding, and endoplasmic reticulum stress, as well as vision-specific processes such as constitutive phototransduction activity and activation of rhodopsin’s cognate G-protein, transducin (21, 22). Notably, there are fewer rhodopsin mutants that are linked to autosomal recessive RP (arRP), including two nonsense mutants (W161X and E249X), and two missense mutants (E150K and M253I) (21). In each of these cases, heterozygous carriers of the respective mutations appear to be mostly normal with little or no visual deficits. The relative abundance of adRP mutations compared to arRP mutations suggests that rhodopsin is highly sensitive to mutation, and mutations in rhodopsin tend to be pathogenic.

No treatments are currently available for *RHO*-associated RP. As *RHO* mutations are responsible for a large proportion of inherited retinal diseases, there has been great interest in developing genetic therapies for halting or reversing rhodopsin-mediated degeneration. These approaches include gene-replacement therapy, although this approach proves to be a particular challenge with dominant mutations, as simple augmentation of the wild-type (WT) gene will not suppress the dominant-negative allele (23). An alternative approach is the use of gene-editing to correct the mutation in vivo, though correction of a dominant allele may still require close to 100% efficiency to prevent cell loss (24). Another gene-editing approach called “knockout and replace” utilizes AAVs to disrupt both WT and mutant genomic *Rho* alleles and simultaneously provide a replacement WT *Rho* complementaryDNA (cDNA). Multiple approaches can achieve this goal, either by using a short hairpin RNA to knock down *Rho*, CRISPR/Cas9 to disrupt endogenous *Rho,* or by using CRISPR/Cas13 to knock down mutant alleles by RNA editing (25–27). While this approach is mutation-independent, substantial challenges include efficacy of knockout, stoichiometry, and control of *Rho* expression, which is toxic if overexpressed, or potentially ineffective if underexpressed. Additionally, the use of nucleases that function by creating double-strand DNA breaks (DSBs) results in highly heterogeneous mixtures of potentially toxic indel outcomes (28), adeno-associated virus (AAV) integration into the genome (29–31), chromosomal abnormalities, and other undesired cellular consequences of DSBs. Last, disruption of one or both alleles responsible for recessive diseases is unlikely to result in significant therapeutic benefit.

To study gene-editing strategies for the treatment of arRP, we used the *Rho*-E150K knock-in mouse model of arRP (32). This rare rhodopsin mutation was reported to cause arRP in three families (33–36). Knock-in *Rho*-E150K mice were generated to study the mechanisms of rhodopsin-mediated retinal degeneration in an autosomal recessive manner, though it was noted that in contrast to human patients, the heterozygous mouse exhibited a mild and delayed retinal degeneration (32). However, we reasoned that in an autosomal recessive disease such as E150K-arRP, therapeutic rescue could be achieved from reasonably efficient editing, and that in a clinical setting, correction of a subset of rod photoreceptors should arrest the progression of retinal degeneration and provide some benefit to patients.

In this study, we employed CRISPR/Cas9-derived precision base editing strategies (37) to correct the rhodopsin-E150K mutation in vivo without requiring double-stranded DNA breaks. Because of bystander editing near the target adenine, we expressed and biochemically characterized all the bystander-edited rhodopsin protein variants. We then demonstrated efficient editing in vivo; however, partial electrophysiological rescue of retinal function was achieved when mice were treated at postnatal day 15 but not at later time points due to progressive retinal degeneration. Histology of treated eyes revealed that in vivo base editing arrested photoreceptor degeneration and preserved rhodopsin expression. In contrast to vision-associated enzymes such as *Rpe65* (38) and *Pde6b* (39), our results suggest that restoration of visual function and retinal structure by gene-editing is more challenging for proteins such as rhodopsin that serve both signaling and structural functions. Nevertheless, with careful application and optimization of factors including treatment timing, gene-editing approaches hold promise as future treatment strategies for *RHO-*associated RP.

## Results

### Development of an Adenine Base Editing (ABE) Strategy for Rho-E150K.

**Fig. 1.**
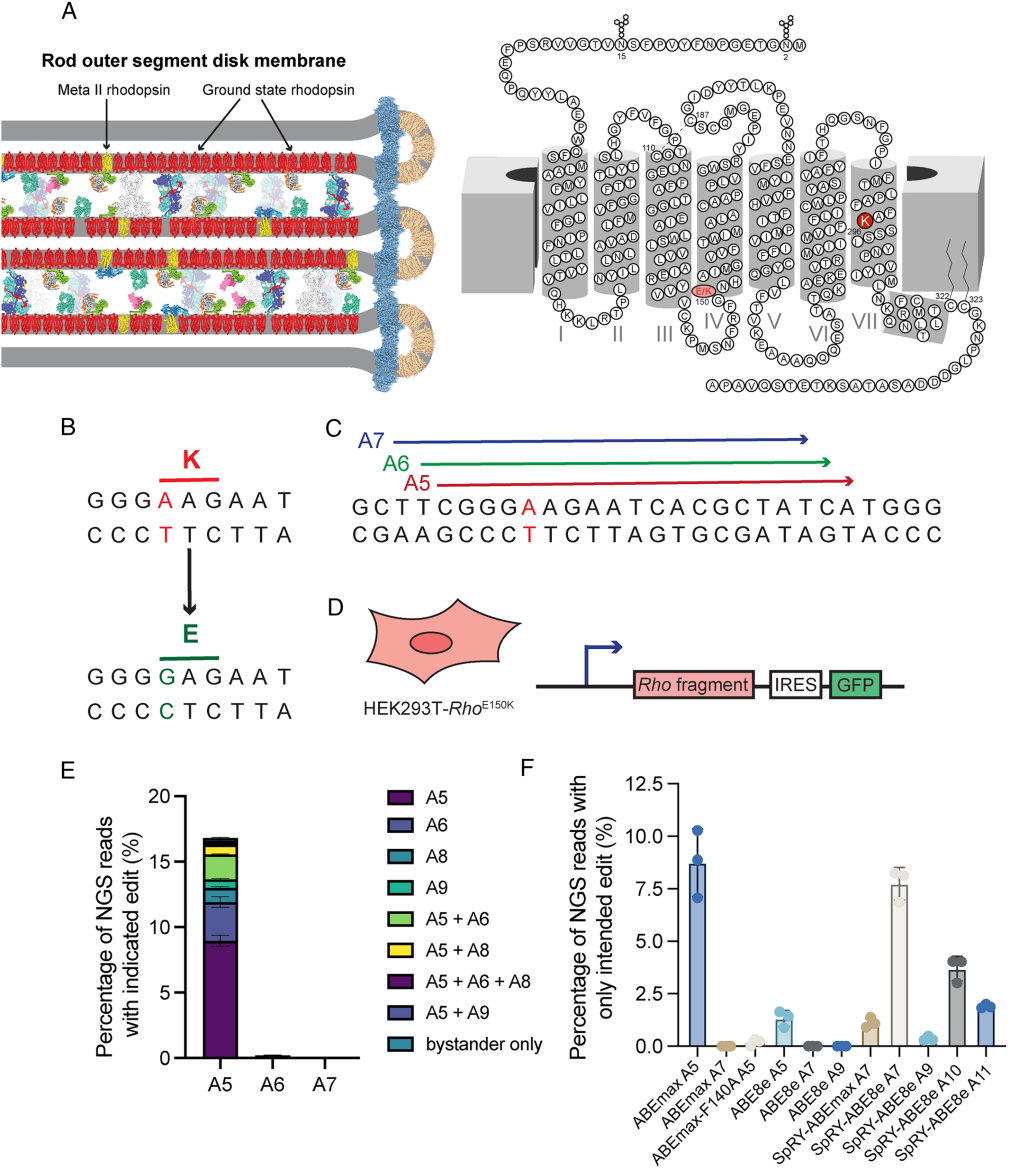
Establishment of rhodopsin-E150K cell line and base editing screening in vitro. (*A*, *Left*) The ROS is composed of stacked disc membranes with a high concentration of rhodopsin (red) (adapted from Gulati and Palczewski) (1). When rhodopsin encounters photons of light, it is photoactivated (yellow) and initiates phototransduction. (*A*, *Right*) 2-D protein structure of mouse rhodopsin. Locations of Schiff-base Lys^296^ and E150K mutation are indicated in red. (*B*) Coding sequence of the E150K-rhodopsin mutation. (*C*) Design of base editing SpCas9 sgRNAs, each named for the location where the adenine of interest is positioned within the protospacer. (*D*) Schematic diagram of the HEK293T-Rho^E150K^ (HEK-E150K) cell line generated by retroviral transduction, used for in vitro screening. IRES-GFP downstream of the *Rho* fragment enables FACS purification of transduced cells. (*E*) Base editing outcomes via next-generation sequencing (NGS) after cotransfection of HEK-E150K cells with plasmids expressing ABEmax and sgRNAs. (*F*) Base editing outcomes via NGS after cotransfection of HEK-E150K cells with various ABEs and sgRNAs with the target adenine placed at positions 5, 7, 9, 10, or 11 within the protospacer.

Rhodopsin is a key structural and sensory protein within the rod photoreceptor. The majority of rhodopsin protein is found within the ROS (Fig. 1*A*). Mutations within rhodopsin are linked to several inherited retinal degenerations. Previously, we generated and characterized a mouse model for *Rho*-E150K autosomal-recessive RP (32). A single-nucleotide G>A mutation results in the change of Glu^150^ to Lys^150^ on the intracellular side of transmembrane helix IV of the protein (c.448G>A, p.E150K) (Fig. 1*A*). This G>A mutation could be addressed by CRISPR/Cas9-derived ABE to revert Lys^150^ to the WT Glu^150^ (Fig. 1*B*). Accordingly, we screened three single-guide RNAs (sgRNAs), placing the target mutated adenine at position 5, 6, or 7 in the protospacer within the activity window of ABEmax (sgRNA A5, A6, and A7, respectively) (Fig. 1*C*). To facilitate screening of ABE strategies to correct the Rho-E150K variant, we created a HEK293T cell line which carries a fragment of *Rho* DNA, termed HEK-E150K (Fig. 1*D*). When we cotransfected WT *Streptococcus pyogenes* Cas9-ABEmax and the A5, A6, and A7 sgRNAs, we noted that only A5 resulted in productive editing, with an average of 12% total editing of the on-target base (on-target plus bystander) and 9.0% precise correction (on-target single-base editing only) (Fig. 1*E*). We also investigated the use of alternative ABE variants, such as ABE8e, SpRY-ABEmax, and SpRY-ABE8e, along with alternative sgRNAs that placed the target adenine at positions 5, 7, 9, 10, and 11 in the protospacer, but none of these combinations matched the precision or efficiency of ABEmax in combination with the A5 sgRNA (Fig. 1*F* and *SI Appendix*, Fig. S1).

### Expression and Characterization of Rho Base Editing Variants.

**Fig. 2.**
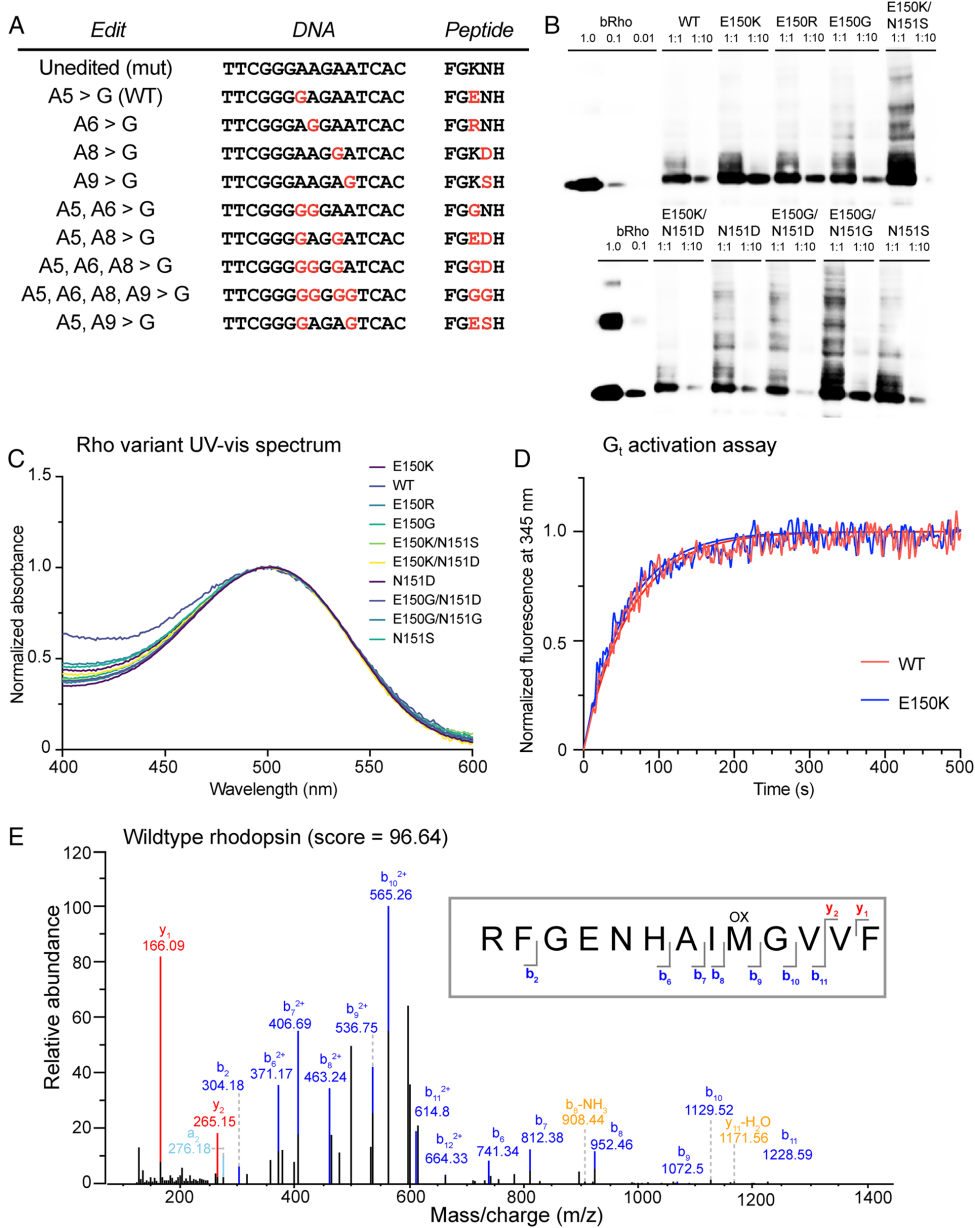
Characterization of bystander-edited variants of E150K-rhodopsin. (*A*) List of all potential coding variants of rhodopsin resulting from on-target and bystander editing observed by next-generation sequencing. (*B*) Anti-1D4 Western blot of HEK293T cells transfected with expression plasmids for all rhodopsin variants, loaded either undiluted (1:1) or diluted (1:10). Purified bovine rhodopsin is used as a positive control (concentration indicated in µg/mL). (*C*) UV-vis absorbance spectra of purified rhodopsin variants in LMNG after reconstitution with 11-*cis*-retinal. Absorbances are normalized to the absorbance of rhodopsin at its λ_max_, 500 nm. (*D*) G_t_ activation assay of WT rhodopsin and the E150K mutant. The results are plotted as normalized increase of fluorescence intensity at 345 nm of G_t_ upon addition of GTPγS. The curves represent the fitting of a pseudo-first-order association model. In this experiment, the rate constants (turnover numbers) were determined to be 16.2 ± 0.4 × 10^−3^ s^−1^ for WT and 17.8 ± 0.4 × 10^−3^ s^−1^ for the E150K mutant. (*E*) Tandem MS/MS spectrum of a unique peptide from purified WT-rhodopsin and its fragmentation pattern.

Our ABE strategy revealed that in addition to target base editing by ABEmax, additional editing outcomes arose from bystander editing of other adenines besides the target adenine within the activity window of ABEmax. The resulting bystander-mutated rhodopsins might lead to altered retinal function (Fig. 2*A*). Therefore, we generated mammalian expression vectors of WT rhodopsin, E150K-rhodopsin, and eight other rhodopsin variants that were observed as editing by-products in the in vitro editing transfection experiments, and expressed them in HEK293T cell culture (Fig. 2 *A* and *B*). When the 10 rhodopsins were reconstituted with 11-*cis*-retinal and purified, the UV-vis absorbance spectra of all 10 exhibited rhodopsin’s characteristic λ_max_ at 500 nm, indicating successful reconstitution of the rhodopsin with its chromophore (Fig. 2*C*). We also assessed the ability of the photoactivated rhodopsin to activate transducin (G_t_) with a fluorescence assay that measures the increase of intrinsic-fluorescence of tryptophan (Trp) in G_tα_ upon binding of the slowly hydrolyzed GTP analogue guanosine 5'-O-[gamma-thio]triphosphate (GTPγS). The kinetic data are represented in Fig. 2*D* for mRho WT and for the mutant E150K, and the rate constant for WT is compared with those for all nine mutants in *SI Appendix*, Table S3. The results suggest that all of the mutants characterized are functional and able to activate transducin in a manner similar to that of WT mRho, consistent with previously reported measures of E150K and WT mRho (40). Last, we characterized and confirmed the identities of the rhodopsin variants by tandem mass spectrometry (MS/MS) (Fig. 2*E* and *SI Appendix*, Fig. S2).

### Development of a Prime Editing Strategy for Rho-E150K.

Our in vitro biochemical characterization of the bystander-edited rhodopsin variants indicated that bystander editing would not affect reconstitution with visual chromophore or G-protein signaling. If behavior of these mutant rhodopsins were different in vivo, a more precise editing strategy that avoids bystander editing would be required. Accordingly, we developed a prime editing (PE) strategy to correct the E150K mutation. We first chose to screen prime editing guide RNAs (pegRNAs) and nicking-guide RNAs (ngRNAs) with PE-SpRY, as previously demonstrated in a *Pde6b* PE strategy (41). We screened 14 pegRNAs targeting spacers around the intended editing site, with a 13-nucleotide primer-binding sequence (PBS) and a 13-nucleotide reverse-transcriptase template, along with six ngRNAs. Our editing outcomes in vitro indicated that several combinations of pegRNA and ngRNA resulted in precise correction (*SI Appendix*, Fig. S3*A*). We opted to further optimize pegRNA-5 in combination with ngRNA-4. We determined that a RTT length of 13 nucleotides and a PBS length of 12 nucleotides was optimal for maximizing editing (*SI Appendix*, Fig. S3 *B* and *C*). However, because evolved Cas9 variants can suffer from decreased efficiency compared to WT Cas9, we also investigated the use of PEmax with a WT Cas9. We used a machine-learning tool (PRIDICT) (42) to design a pegRNA and noted that editing efficiency with PEmax was substantially higher with the PRIDICT pegRNA compared to the PE-SpRY editor with optimized p5, both with and without ngRNA-4: PE-SpRY reached 10.4% editing without ngRNA-4 and 16.4% editing with ngRNA-4, PEmax achieved 13.6% editing without ngRNA-4 and 20.8% editing with ngRNA-4 (*SI Appendix*, Fig. S3*D*). PEmax editing also resulted in lower indel formation than PE-SpRY in combination with ngRNA-4, with indel rates of 3.1% versus 6.4%, respectively (*SI Appendix*, Fig. S3*D*). While more recent developments in PE systems would likely increase the efficiency of PE outcomes and enable in vivo PE, including a dual-AAV system to deliver PEmax that was developed after the in vitro and in vivo BE studies described here (43–47), given the high efficiency of base editing and the data above suggesting that the observed *Rho* bystander base editing outcomes did not abrogate rhodopsin function, we advanced our base editing strategy into studies in vivo.

### Base Editing of Rhodopsin in arRP Mice.

**Fig. 3.**
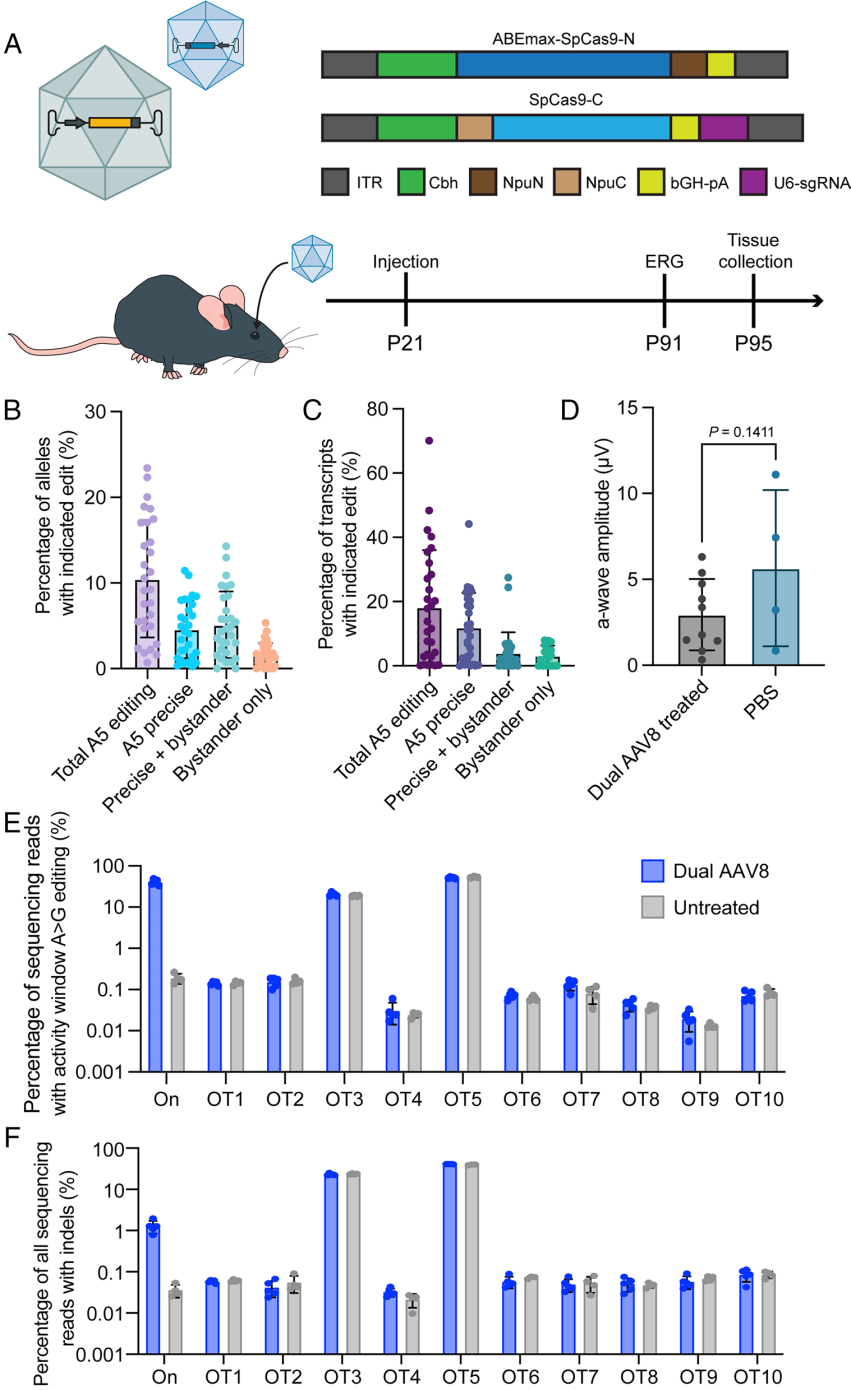
In vivo ABE of *Rho*-E150K mice treated at P21. (*A*) Schematic diagram (*Upper*) depicting the dual-AAV strategy for in vivo base editing. The gene coding for ABEmax and the sgRNA are split into a N-terminal and a C-terminal AAV. When a cell is transduced by both AAVs, the full-length ABEmax is reconstituted via Npu intein splicing. Schematic diagram (*Lower*) illustrating the P21 animal-treatment protocol: E150K mice were treated at postnatal day 21 (P21) by subretinal injection of dual-AAV8 and analyzed by ERG 10 wk later (P91). Retinas were then collected and analyzed by NGS (51). (*B*) NGS analysis of bulk retinal genomic DNA for base editing outcomes after dual-AAV8 treatment. (*C*) NGS analysis of bulk retinal cDNA (synthesized from retinal RNA) for base editing outcomes after dual-AAV8 treatment. (*D*) ERG a-wave amplitudes from E150K mice after dual-AAV8 treatment, compared with E150K mice treated with PBS (controls). *P* = 0.1411 by Student’s *t* test. (*E*) CIRCLE-seq analysis of A>G editing of on- and off-target genomic DNA within the ABE activity window (protospacer positions 4 to 8) for retinas from E150K dual-AAV8-treated mice versus untreated mice. (*F*) CIRCLE-seq analysis of all indels within the entire NGS amplicon for retinas from E150K dual-AAV8-treated mice versus untreated mice. All results are represented as mean ± SD.

As demonstrated in other studies targeting photoreceptors, we generated dual-AAV vectors that each encoded part of ABEmax fused to N- and C-terminal inteins to mediate restoration of full-length base editor after dual transduction (48). We produced two AAV vectors with Cbh promoters driving N-terminal ABEmax on one genome and C-terminal ABEmax on a separate genome, which also contains a U6 sgRNA expression cassette (Fig. 3*A*). These genomes were then packaged into AAV2/8 viral vectors. To perform base editing in vivo, we injected a 1:1 mix of the dual-AAV8s subretinally into homozygous *Rho*-E150K mice at postnatal day 21 and assessed electroretinography (ERG) and sequencing data 10 wk postinjection (Fig. 3*A*). We reasoned that this posttreatment period would allow sufficient time for AAV expression, editing, and assessment of phenotypic rescue. At 10 wk postinjection, we collected neural retinas from dual-AAV8-treated animals. Along with genomic DNA, we also collected RNA from the retinas. While there are over 100 other cell types found within the retina, *Rho* is expressed only within rod photoreceptors, so analysis of both genomic DNA and complementary DNA (cDNA) synthesized from RNA enabled us to assess editing outcomes in our cell type of interest (49). In the genomic DNA, we noted an average of 10.5% total editing at the on-target base, with an average of 4.6% precise single-base correction, and a maximal precise correction rate of 11.4% (Fig. 3*B*). In the cDNA, the average of total editing of the on-target base was 18.2%, with an average of 11.9% precise correction, and a maximum precise correction rate of 44.2% (Fig. 3*C*). However, we did not notice a phenotypic rescue of the ERG a-wave, attributable to photoreceptor activation, as ERG a-wave amplitudes were lower in dual-AAV8-treated animals compared to a phosphate-buffered saline-injected control (Fig. 3*D*). Last, to assess off-target effects of ABEmax, we performed CIRCLE-seq analysis (50) of retinas from treated and untreated *Rho*-E150K mice. We noted no difference in indels or A>G base editing within the activity window, or indels at the top 10 nominated sites (Fig. 3 *E* and *F*).

**Fig. 4.**
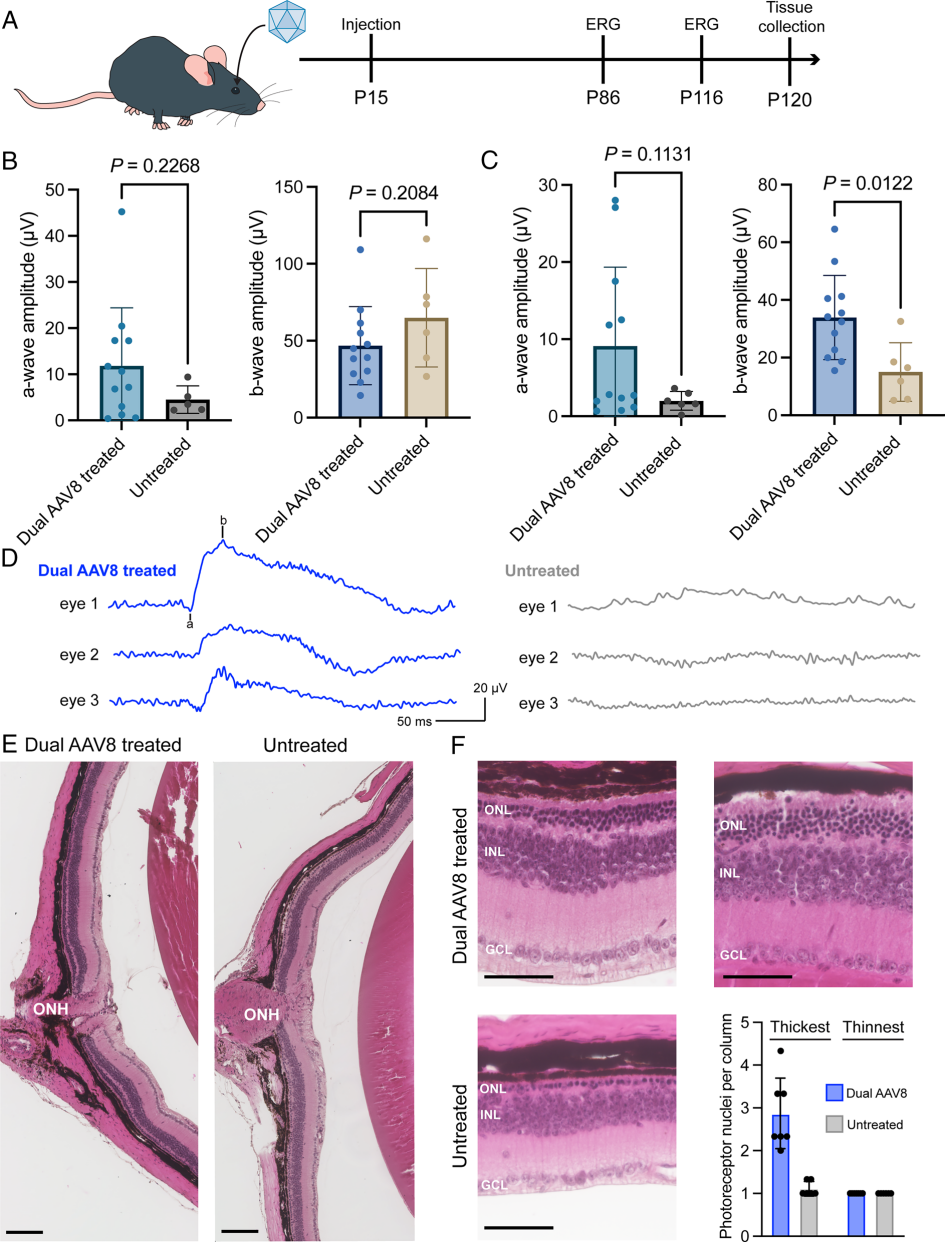
In vivo ABE of *Rho*-E150K mice treated at P15. (*A*) Schematic diagram of P15 treatment protocol: E150K mice were treated at postnatal day 15 (P15) by subretinal injection of ABE-expressing dual-AAV8, and analyzed by ERG after 10 wk (P86) or 14 wk (P116). Retinas were then collected and analyzed by NGS. (*B*) ERG a-wave (*Left*) and b-wave (*Right*) amplitudes from E150K mice 10 wk after dual-AAV8 treatment. *P* = 0.2268 and 0.2084 by Student’s *t* test. (*C*) ERG a-wave (*Left*) and b-wave (*Right*) amplitudes from E150K mice 14 wk after dual-AAV8 treatment. *P* = 0.1131 and 0.0122 by Student’s *t* test. (*D*) Representative ERG waveforms at −0.3 log cd s m^−2^ from three dual-AAV8-treated mice (*Left*) and three untreated mice (*Right*). The ERG a- and b-wave markers are indicated on trace 1 (treated mice). (*E*) Representative hematoxylin and eosin sections from dual-AAV8-treated mice (*Left*) and untreated mice (*Right*). ONH, optic nerve head. Scale bar represents 100 µm. (*F*) Representative hematoxylin and eosin sections from the thickest region of the retina from dual-AAV8-treated and untreated mice. Quantification of photoreceptor nuclei per ONL, from thickest and thinnest regions of retinas. Scale bar represents 50 µm. ONL, outer nuclear layer; INL, inner nuclear layer; GCL, ganglion cell layer. All results are represented as mean ± SD.

In conjunction with our biochemical characterization of rhodopsins, we had hypothesized that our editing rates would have been sufficient to rescue the degeneration phenotype. We reasoned that the incomplete phenotypic rescue could be due to a delay in the timing of editing relative to what would be required for functional rescue. We previously reported that untreated homozygous *Rho*-E150K mice had already lost >20% of their photoreceptors at postnatal day 30, and 2-mo-old homozygous *Rho*-E150K mice were >60% degenerated (32). Therefore, we next treated homozygous *Rho*-E150K mice 6 d earlier at postnatal day 15 with the same dual-AAV8 strategy (Fig. 4*A*). 10 wk postinjection, we noted a trend toward rescue of the ERG a- and b-wave phenotypes with higher amplitudes recorded from dual-AAV8-treated mice compared to untreated controls (Fig. 4*B*). Therefore, we remeasured ERG amplitudes at 14 wk postinjection, at which untreated homozygous mice have lost nearly all photoreceptors and ERG responses, while a rescue of the phenotype would result in preservation of the ERG wave (32). As we predicted, the treated animals retained some of the ERG a- and b-wave, while the untreated animals continued to degenerate (Fig. 4 *C* and *D*). To examine the anatomical rescue of the retinas, we assessed eyes from dual-AAV8-treated mice and untreated controls along the superior–inferior axis to measure the outer nuclear layer (ONL) where the photoreceptor nuclei reside (Fig. 4*E*). As the gene-editing therapy may not evenly treat the entire retina, we evaluated the entire retina and quantified the number of photoreceptor nuclei at the thickest and thinnest points of each retina. We found that treatment with dual-AAV8 led to preservation of the ONL, with a greater number of photoreceptor nuclei per column in the thickest part of each retina, whereas the thinnest part of the retina, likely untreated by dual-AAV8, did not show a difference in the photoreceptor nuclei count (Fig. 4*F*).

**Fig. 5.**
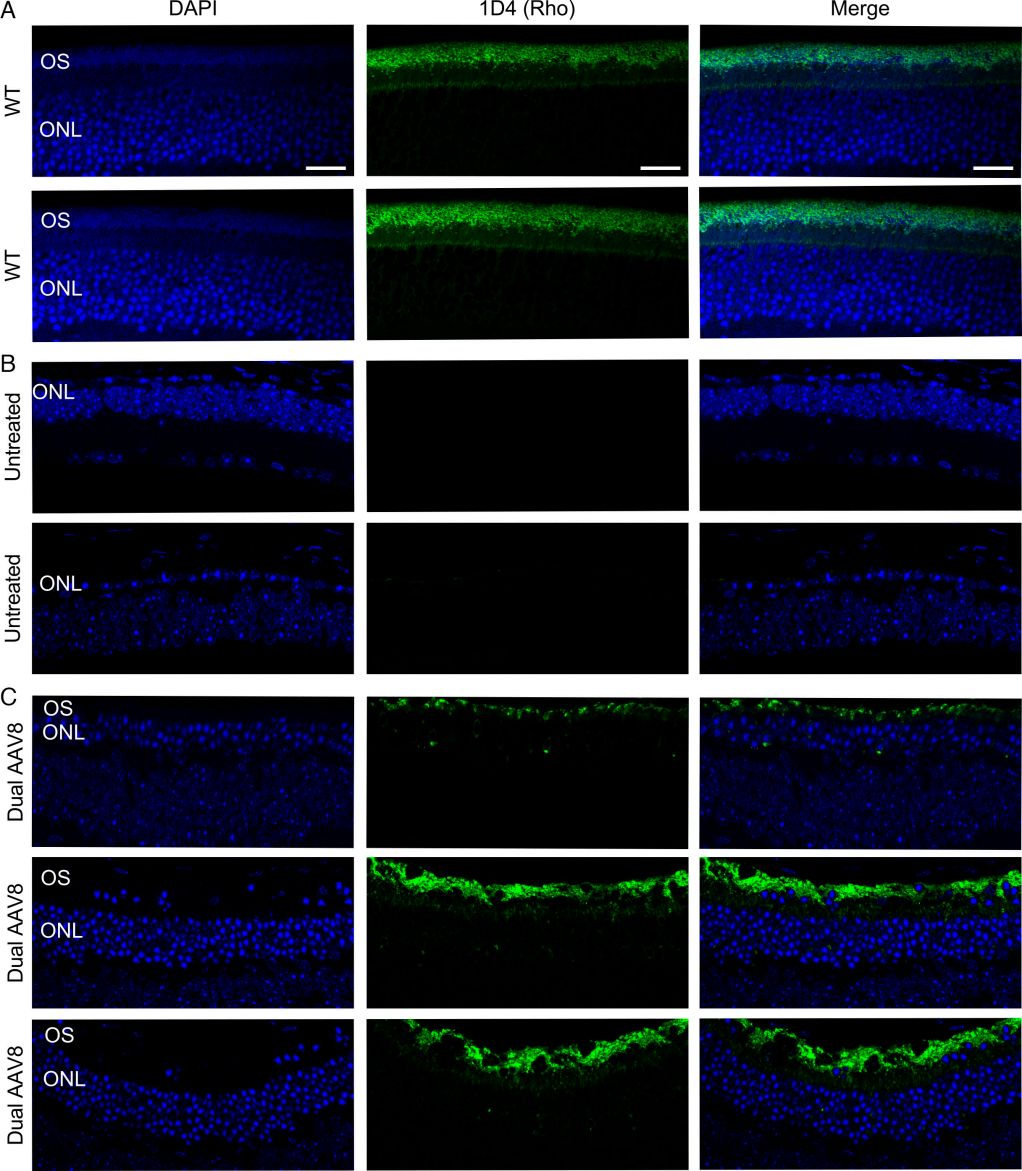
Rhodopsin expression in E150K mice after base editing at P15. Representative immunohistochemistry of retinal sections stained with DAPI and 1D4 (anti-Rho) of (*A*) WT, (*B*) untreated *Rho*-E150K mice, and (*C*) *Rho*-E150K mice treated with ABE-expressing dual-AAV8 at P15. Each row represents an individual and independent eye taken from mice 15 wk posttreatment or the equivalent age for WT and untreated mice. ONL, outer nuclear layer; OS, outer segment. Scale bar represents 20 µm.

To confirm that we restored rhodopsin expression in dual-AAV8-treated mice, we performed immunohistochemistry 15 wk posttreatment on age matched WT mice, untreated *Rho*-E150K mice, and *Rho*-E150K mice treated with dual-AAV8 at P15. Staining with DAPI and 1D4 revealed robust rhodopsin expression in the outer segment of retinas from WT mice that was absent in the retinas from untreated E150K mice, while dual-AAV8 treatment restored rhodopsin expression in the retinas of treated E150K mice (Fig. 5). Correlating with the expression of rhodopsin, retinas from untreated E150K mice only exhibited one single column of ONL nuclei, while retinas from treated E150K mice exhibited multiple ONL nuclei per column.

## Discussion

In this report, we provide evidence that genetic mutations in rhodopsin can be corrected to provide modest therapeutic benefit in a mouse model of RP. We also demonstrate two precision-editing approaches, ABE and PE. Many previous studies on precision-editing in the mouse retina focused on correction of mutations which affect enzymes, including *Rpe65* (38) and *Pde6b* (41), which catalyze biochemical reactions critical for light detection and phototransduction. To our knowledge, fewer studies have focused on proteins that provide structural support to photoreceptors, such as *Cep290* (52); fewer still have investigated proteins such as rhodopsin which provide both structural and signaling properties to the cell. Our study highlights both the promise and potential challenge in reaching a therapeutic threshold for rhodopsin and other structural and signaling proteins in photoreceptors and other cell types.

We showed that rhodopsin could be edited productively, with a maximum of 44% transcript editing. Treatment of E150K mice with dual-AAV8 at postnatal day 15 was able to rescue the ERG photoresponse compared to untreated E150K mice, which did not show the a- or b-waves in response to photostimulation. The dual-AAV8 treatment also prevented the complete loss of photoreceptor nuclei in the ONL. In the untreated animals, the remaining cells in the ONL did not express rhodopsin; thus, likely they are residual cone photoreceptors. Cone photoreceptors have been shown to persist in patients with RP and mouse models of RP after the death of rod photoreceptors, though the surviving cone bodies are often dysfunctional and eventually die (53, 54). As rod photoreceptors support the survival of cone photoreceptors, which mediate high-acuity daytime vision, the rescue of rod photoreceptors is expected also to improve cone function and maintain vision for RP patients. While we did observe a preserved ERG response in the treated animals, the amplitudes were modest compared to WT. This is likely due to the fact that the rescue was uneven across the retina, which some areas of treated retinas demonstrating strong rescue, while other areas of treated retinas showing no rescue (Fig. 4*F*). Further optimization of dosing and surgical technique could increase the total rescued area in mice or in patients.

Based on the known volume and concentration of rhodopsin within the ROS, it is possible to calculate the number of rhodopsin molecules per rod photoreceptor, which is approximately 6.4 × 10^7^ (11, 55, 56). In the mammalian retina, roughly 10% of the ROS is turned over every day; thus, 10% of the rhodopsin content must be replaced every day. As each molecule of mRNA, on average, creates between 2,800 and 4,200 protein molecules (57, 58), each rod photoreceptor generates roughly 3,400 to 5,000 molecules of *Rho* messenger RNA (mRNA) per day, or at least one *Rho* mRNA molecule every 25 s; thus, for a mouse retina with 6.4 million rods, about 250,000 mRNA molecules are generated every second. An alternative approach to estimating the rhodopsin concentration in the eye is by analysis of its chromophore, 11-*cis-*retinal, which in a dark-adapted mouse retina amounts to at least 500 pmol, nearly all bound to rhodopsin stoichiometrically (59, 60). This calculation estimates at least 3 × 10^14^ rhodopsin molecules, within a factor of three of our calculation above [(1.4 × 10^8^ per rod) × (6.4 × 10^6^ rods) = 9 × 10^14^ Rho molecules] (11, 61). These estimates indicate that the phenotypic changes observed are a result of significant alterations of *Rho* transcript and protein production that persists posttreatment. Indeed, as heterozygous *Rho*-E150K mice exhibit altered ROS morphology, ROS number, and phototransduction kinetics, alterations in *Rho* expression can lead to dramatic changes in visual physiology (11). Edited genomic DNA would be expected to provide sufficient WT rhodopsin within 2 wk of successful editing, though we only noted significant phenotypic rescue after treatment at postnatal day 15 and not at postnatal day 21. This timing difference may be due to the kinetics of ABE expression. AAV8 has been shown to express as soon as 7 d postinjection, though maximal expression of the cargo occurs around 30 d postinjection (62). In animal models and patients where degeneration begins early and progresses quickly, the kinetics of gene editor delivery and treatment may play a crucial role. Thus, earlier diagnosis and treatment could be critical for therapeutic benefit in cases where the degeneration and structural loss is relatively rapid.

Knowledge of the mechanisms of retinal degeneration in E150K-associated arRP and other forms of arRP remains limited. It has been hypothesized that the E150K mutation impacts protein trafficking and higher-order oligomerization of rhodopsin in the outer segment, and we reasoned that nonproductive or bystander editing could still disrupt ROS organization; however, our biochemical characterization of bystander-edited rhodopsin variants showed that they remained functional, so elimination of the E150K mutation by on-target or bystander editing could restore proper dimerization. It is also possible that the bystander-edited mutants were unable to traffic correctly to the plasma membrane due to a disruption of electrostatics that need to be compensated, similarly to the E150K variant previously characterized in vitro (63). With the exception of the A6 bystander edit (Lys^150^ to Arg^150^), productive or bystander editing results in the removal of the positive charge on Lys^150^. This could be sufficient to prevent degeneration in the E150K mouse, as we had previously proposed that Lys^150^ disrupts higher-order packing and multimerization in the mouse ROS (32). Thus, future in vivo studies utilizing the PE approach proposed here could result in therapeutic benefit to eliminate the possibility of bystander editing causing de novo or unarrested degeneration (*SI Appendix*, Fig. S3). Though heterozygous carriers of *Rho*-E150K did not exhibit any retinal abnormalities, heterozygous mice exhibited delayed retinal degeneration (32). This delayed degeneration could result from differences in the physiology and structure of the mouse proteins and cells and may indicate that a greater fraction of accurate-editing outcomes may be required to rescue the homozygous-mouse Rho-E150K phenotype relative to the threshold potentially required for humans.

The *RHO*-E150K and other mutations are individually rare, so it may be difficult to bring a candidate therapy through clinical trials and approval. Accordingly, innovative mutation-agnostic editing strategies using a combination of complementary therapies that extend the therapeutic window, as demonstrated for other gene-editing strategies (64, 65), or a simplified regulatory framework (66), could be critical to provide RP patients with a molecularly targeted therapy.

## Methods

### Animals.

Rho-E150K knock-in animals were previously described (32). The animals were housed at the University of California, Irvine, where they were maintained on a unrestricted regular diet and a 12 h light/12 h dark cycle. Mouse experiments were approved by the Institutional Animal Care and Use Committee of the University of California, Irvine, were performed in accordance with the NIH Guidelines for the Care and Use of Laboratory Animals, and with the ARVO Statement for the Use of Animals in Ophthalmic and Visual Research.

### Expression and Purification of mRho Variants.

To express mRho variants, HEK293T/17 cells were seeded into 15-cm tissue-culture dishes the day before transfection. 20 µg of plasmid was transfected into cells via PEI max (3:1 ratio PEI:plasmid complexed in OptiMEM, Polysciences #24765-100). 48 h posttransfection, cells were collected by trypsinization and pelleted for protein purification.

Cells expressing rhodopsin mutants were subjected to hypotonic shock with 10 mM N-2-hydroxyethylpiperazine-N-2-ethane sulfonic acid (HEPES pH 7.5) plus protease inhibitor cocktail (Roche), 1 mM MgCl_2_, and benzonase. After centrifugation, the pellet was resuspended in 10 mM HEPES pH 7.5, 0.25 M NaCl plus protease inhibitor cocktail (Roche), 1 mM MgCl_2_, and benzonase. Then, each sample was incubated in the dark with 90 µM of 11-*cis-*retinal for 15 min at room temperature. Next, the membranes were solubilized in 6 mM lauryl maltose neopentyl glycol (LMNG) for 2 h at 4 °C and centrifuged at 21,000 × g for 20 min. Then, rhodopsin in the supernatant was immunopurified using immobilized 1D4 antibody as previously described (67, 68). In brief, solubilized membranes were incubated for 2 h with the 1D4-Sepharose, and then the medium was washed with 40 column volumes of washing buffer (0.2 mM LMNG in 20 mM HEPES, pH 7.4, 0.15 M NaCl). The rhodopsin mutants were then eluted by competition with 1 mg/mL peptide TETSQVAPA in washing buffer.

### G_t_ Activation assay.

Activation of transducin (G_t_) was assessed by the increase in intrinsic tryptophan fluorescence upon nucleotide exchange catalyzed by photoactivated Rho. G_t_ was extracted from frozen bovine ROS membranes as described elsewhere (69, 70). The time course of intrinsic-fluorescence change from G_tα_ was measured with an L55 luminescence spectrophotometer (PerkinElmer Life Sciences) operating at excitation and emission wavelengths of 300 and 345 nm, respectively. Rho (25 nM) was mixed with G_t_ (500 nM) in 20 mM bis-tris propane pH 7.0, 120 mM NaCl, 2 mM MgCl_2_, 0.5 mM LMNG. Then, Rho was photoactivated for 30 s with 505-nm fiber light (625 μW) and the fluorescence emission was measured for 5 min. This was followed by the addition of 5 µM GTPγS to induce Rho/G_t_ complex dissociation and increase in G_tα_ fluorescence. The pseudo-first-order rate constants (*k*) of G_t_ activation were determined from the first 1,500 s of the assay.

### In Vitro Plasmid Transfection and Cell Culture Genomic DNA Isolation.

The day before transfection, HEK293T-Rho^E150K^ cells were seeded into 48- or 96-well plates to achieve ~70% confluency on the day of transfection. Plasmids were transfected with Lipofectamine 3000 (Thermo #L3000001) according to manufacturer instructions. 48 h posttransfection, cells were lysed as previously described (71). Briefly, culture medium was removed and the cells were incubated in lysis buffer [10 mM pH 8 Tris-HCl, 0.05% sodium dodecyl sulfate, 1:1,000 proteinase K (New England Biolabs #P8107S)] at 37 °C for 1 h. Then, the proteinase K was inactivated by incubation at 80 °C for 30 min. Crude lysates were stored at −20 °C.

### AAV Preparation.

AAV2 genomes were generated as previously described (48) and propagated in Stbl3 *Escherichia coli* (Thermo #C737303). AAVs were packaged by SignaGen Laboratories (Frederick, MD) into AAV8 capsids at a final titer of 1.53 × 10^13^ genome copies (GC) mL^−1^ (N terminus) and 1.72 × 10^13^ GC mL^−1^ (C terminus). AAVs were mixed at a 1:1 particle ratio and stored at −80 °C until injection. Mixed AAVs were injected in a 1 µL solution, containing 7.65 × 10^9^ total GC each of N and C terminus AAVs.

### Subretinal Injections.

Mice were bilaterally dilated, first with topical administration of 1% tropicamide ophthalmic solution (Akorn, 17478-102-12), followed by 10% phenylephrine ophthalmic solution (MWI Animal Health, 054243). Mice were then anesthetized by intraperitoneal administration of 20 mg mL^−1^ ketamine and 1.60 mg mL^−1^ xylazine in phosphate buffered saline (PBS) at a dose of 100 mg kg^−1^ of ketamine and 8 mg kg^−1^ of xylazine. To maintain corneal hydration, a drop of GenTeal Severe Lubricant Eye Gel was applied (0.3% hypromellose, Alcon). Subretinal injections were performed under an ophthalmic surgical microscope (Zeiss). Using a 27G beveled needle, an incision was made in the cornea proximal to the limbus at the nasal side. A 34G needle with a blunt tip (World Precision Instruments, NF34BL-2), connected to an Nanofil injection holder (World Precision Instruments, NFINHLD) with SilFlex tubing (World Precision Instruments, SILFLEX-2), was inserted through the corneal incision into the anterior chamber and advanced into the subretinal space without touching the lens. Each mouse received a 1 μL injection in each eye at 70 nL s^−1^, controlled by a UMP3 UltraMicroPump (World Precision Instruments, UMP3-4). After surgery, the mice were placed on a heating pad and anesthesia was reversed with intraperitoneal 2.5 mg kg^−1^ atipamezole in PBS, (MWI Animal Health, #032800). Triple antibiotic ophthalmic ointment (neomycin, polymyxin, and bacitracin) was administered to the cornea to promote recovery.

### ERG.

Prior to ERG recording, mice were dark-adapted overnight. Under a safety light, mice were bilaterally dilated, first with topical administration of 1% tropicamide ophthalmic solution (Akorn, 17478-102-12), followed by 10% phenylephrine ophthalmic solution (MWI Animal Health, 054243). Mice were then anesthetized by isoflurane inhalation. To maintain corneal hydration, a drop of GenTeal Severe Lubricant Eye Gel was applied (0.3% Hypromellose, Alcon). The mouse was placed on a Diagnosys Celeris rodent-ERG device preheated to 37 °C (Diagnosys LCC, Lowell, MA, USA). Ocular stimulator and recording electrodes were placed to cover the corneas, a reference electrode was placed subdermally between the ears, and a ground electrode was positioned subdermally in the left rear thigh. The mice were stimulated with 544 nm light at an intensity of −0.3 log (cd s m^−2^) (160 nm bandwidth). Voltage recordings for 10 repeated stimuli, with 10 s between each stimulus, were combined to form the averaged ERG waveform in Espion V6 software (Diagnosys LLC). A- and b-wave amplitudes and annotations were verified before analysis.

### Retina Dissociation and Genomic DNA and Total RNA Isolation.

Mouse eyes were dissected under a light microscope to remove the anterior segment. Then, the neural retina was then separated from the RPE, choroid, sclera. Each retina was immediately immersed in RLT-Plus (Qiagen), disrupted with a motorized pestle (Fisher #12-141-361), and then homogenized in a QiaShredder (Qiagen # 79656). The lysate was then processed for genomic DNA and RNA with the AllPrep DNA/RNA Micro kit according to manufacturer instructions (Qiagen #80284).

### Next-Generation Sequencing.

cDNA was synthesized from RNA with the SuperScript III First-Strand Synthesis SuperMix (Thermo Fisher #18080400), according to the manufacturer’s instructions. 0.5 to 1 μL of the isolated genomic DNA or cDNA was used as input for the first of two PCRs (PCR1). Genomic loci were amplified in PCR1, using Phusion Plus polymerase (Thermo Fisher Scientific F631S). PCR1 primers for genomic loci are listed in *SI Appendix*, Table S1. PCR1 was performed as follows: 98 °C for 30 s; 30 cycles at 98 °C for 10 s, 60 °C for 20 s, and 72 °C for 30 s; 72 °C for 5 min. PCR1 products were confirmed on a 1% agarose gel. One microliter of PCR1 was used as input for PCR2 to install Illumina barcodes. PCR2 was conducted for nine cycles of amplification using a Phusion HS II kit (Life Technologies). Following PCR2, samples were pooled, and gel-purified in a 1% agarose gel using a Qiaquick Gel Extraction Kit (Qiagen). Library concentration was quantified using the Qubit High-Sensitivity Assay Kit (Thermo Fisher Scientific). Samples were sequenced on an Illumina MiSeq instrument (paired-end read, read 1: 200 to 280 cycles, read 2: 0 cycles), using an Illumina MiSeq 300 v2 Kit (Illumina).

### Histology.

Mice were sacrificed by CO_2_ inhalation before whole eye enucleation. Excess tissues were removed by microdissection before eyes were placed into Hartman’s fixative (Sigma-Aldrich #H0290) and fixed for 24 h at 4 °C. The eyes were then stored in 70% ethanol before paraffin embedding. Sections were cut to 8 µm thickness onto Superfrost Plus slides (Thermo #12-550-15) before hematoxylin and eosin (H&E) staining by standard procedures. Images were acquired on a Keyence BZ-X810 All-in-One fluorescence microscope.

### Immunohistochemistry.

Hartman’s-fixed and paraffin-embedded sections were rehydrated with sequential washes of xylene, ethanol, and PBS before blocking with 5% normal donkey serum (NDS) in 0.2% Triton X-100 in PBS at room temperature for 1 h. Sections were then incubated with primary antibodies with 2.5% NDS in 0.1% Triton X-100 in PBS overnight in the dark at 4 °C (mouse 1D4 anti-rhodopsin-Alexa Fluor 488, 1:500, in-house). The sections were then washed with 0.1% Triton X-100 in PBS at room temperature in the dark three times, with each wash being 10 min, before sections were mounted with VectaShield HardSet Antifade Mounting Medium with DAPI (Vector Labs, #H-1500-10) and a coverslip. Images were acquired on a Leica Stellaris SP8 confocal microscope.

## Supplementary Material

Appendix 01 (PDF)

## Data Availability

High-throughput sequencing files have been uploaded to the NCBI SRA under accession PRJNA1129549 (72). Some study data are available, and all other data necessary to evaluate the conclusions are included in the paper and/or the *SI Appendix*. Any additional data not found within the article or Supplemental Information, as well as novel materials, can be obtained upon reasonable request to the authors.
