# Supplementary material for: In vivo photoreceptor base editing ameliorates rhodopsin-E150K autosomal-recessive retinitis pigmentosa in mice
Source: Proc Natl Acad Sci U S A. 2024 Nov 18;121(48):e2416827121. doi: 10.1073/pnas.2416827121 (PMC11621631; doi:10.1073/pnas.2416827121)
Supplement: Supplementary file 1 — Appendix 01 (PDF) [file pnas.2416827121.sapp.pdf]

SUPPLEMENTARY INFORMATION

***In vivo* photoreceptor base editing ameliorates rhodopsin-E150K autosomal-recessive retinitis pigmentosa in mice**

Samuel W. Du[1,2,*], Gregory A. Newby[3,4,5,6,7], David Salom[1], Fangyuan Gao[1], Carolline Rodrigues Menezes[1,2], Susie Suh[1], Elliot H. Choi[1], Paul Z. Chen[3,4,5,8,9], David R. Liu[3,4,5,*], Krzysztof Palczewski[1,2,10,11*]

*To whom correspondence should be addressed:

Krzysztof Palczewski, Center for Translational Vision Research, Department of Ophthalmology, Gavin Herbert Eye Institute, UCI, Irvine, CA, 92697; phone (949) 824-6527; kpalczew@uci.edu

David R. Liu, Merkin Institute of Transformative Technologies in Healthcare, Broad Institute of MIT and Harvard, Cambridge, MA; phone (617) 496-1067; drliu@fas.harvard.edu

Samuel W. Du, Center for Translational Vision Research, Department of Ophthalmology, Gavin Herbert Eye Institute, UCI, Irvine, CA, 92697; phone (949) 824-5154; swdu@uci.edu

Supplemental Methods

Supplemental Figure 1. Screening of alternate base-editing strategies *in vitro*.

Supplemental Figure 2. Tandem MS/MS spectra of purified peptides from rhodopsin variants.

Supplemental Figure 3. *In vitro* screening of engineered prime-editing guide RNAs (epegRNAs) for correction of Rho-E150K.

Supplemental Table 1. PCR primers used in this study.

Supplemental Table 2. Key plasmid sequences used in this study.

Supplemental Table 3. Normalized Gt kinetic constants.

Supplemental Table 4. CIRCLE-seq nominated sites.

Supplemental References

Supplemental Methods

Molecular cloning

All plasmids for transfection were prepared by endotoxin-free mini-, midi-, or maxi-prep (Thermo #K210002, K210014, or K210016). To generate hU6-sgRNA expression vectors, oligos corresponding to the protospacer were used to PCR-mutagenize and KLD-clone (NEB #M0554S) the hU6-rd12-A6 sgRNA-expression vector (1). To generate mammalian expression vectors for mouse rhodopsin (mRho), Rho-E150K retinal cDNA was subjected to PCR with primers appending EcoRI and NotI restriction sites. Then, a pcDNA3.1 vector was double digested with EcoRI and NotI, the backbone was purified by QiaQuick MinElute gel extraction (Qiagen #28604), the fragments were assembled by InFusion Gibson assembly (Takara #638945), and the assembly was transformed into Mach1 competent *E. coli* (Thermo #C862003) to generate pcDNA3.1-mRho-E150K. The vector pcDNA3.1-mRho-E150K was then PCR-mutagenized and KLD-cloned (NEB #M0554S) to generate the other 9 mRho variants.

Generation of HEK293T cell line expressing Rho-E150K

A HEK293T cell line stably expressing a fragment of Rho-E150K (HEK-E150K) was generated by transduction of naïve HEK293T cells with helper-free retrovirus collected from Phoenix-Ampho cells transfected with pMXs-E150K-IRES-GFP, as previously described (2). To generate pMXs-E150K-IRES-GFP, a 279bp fragment of the murine *Rho* (rhodopsin) gene was PCR-amplified and then double digested with EcoRI and NotI and inserted into the MCS of pMX-IRES-GFP (a gift from T. Kitamura at the University of Tokyo). Downstream of the *Rho* fragment, the IRES-GFP allows for cell sorting by flow cytometry. GFP+ cells were collected on a FACSAria Fusion cell sorter (BD Biosciences) to enrich transduced cells. Cells were clonally isolated by sequential limiting dilution, and the construct was sequenced. Cells were maintained in DMEM with glutamine (Thermo Fisher, 11965092), 10% FBS (Genesee Scientific, San Diego, CA, USA, 25-514H), and optional 100 U ml^{-1} penicillin-streptomycin (Thermo Fisher, 15140122), in a humidified incubator at 37 °C with 5% CO_2.

Mass spectrometry sample preparation

The purified rhodopsin samples were digested by the filter-aided sample-preparation (FASP) method. Briefly, the sample was diluted with 50 mM NH_4HCO_3 and then transferred into a Amicon Ultra 0.5 spin-filter column (30 kDa). Afterwards, the concentrated proteins were reduced by 10 mM DTT at 56 °C for 1 h and alkylated by 20 mM IAA at room temperature in the dark for 1 h. Then, the protein solution was washed with 50 mM NH_4HCO_3 three times *via* dilution-

concentration cycles. Lys-C was added into the samples from the E150K, WT, E150G, E150K/N151S E150K/N151D, N151D, E150G/N151D, E150G/N151G, and N151S rhodopsin samples, and Arg-C was added into the samples from the E150R rhodopsin sample at a ratio of 1:50 (protease:protein) and incubated at 37 ^{o}C for 4 h. Then, 1 volume of 200 mM Tris-HCl, 20 mM $CaCl_2$ was added before chymotrypsin was spiked at a ratio of 1:50 (protease:protein) and incubated at room temperature overnight. The digests were recovered by centrifugation as flow-through 30-kDa MW-cutoff filters, the filters washed with water, and the filtrates were combined. The resultant supernatant solution was vacuum-dried and then adjusted to 200 μL with 0.5% acetic acid. Then the peptide mixture was subjected to C18 solid-phase extraction (Nest Group, Inc.) for desalting, and subsequently vacuum-dried.

Mass spectrometry data acquisition.

Proteomics data were acquired *via* LC-MS/MS using an UltiMate 3000 UHPLC (Thermo Fisher Scientific) coupled in-line with an Orbitrap Fusion Lumos mass spectrometer (Thermo Fisher Scientific) with an ESI nanospray source. Mobile phase (buffer B) was comprised of 0.1% formic acid in acetonitrile. The total flow rate was 300 nL min^{-1}, and peptides were separated over a 57-min gradient from 4% to 25% buffer B (total run time 90 min per sample) on an Acclaim PepMap RSLC column (50cm x 75 μm). Survey (MS) scans were acquired in Orbitrap (FT) with automated gain control (AGC) target 8E5, maximum injection time 50 ms, and dynamic exclusion of 30 s across the scan range of 375-1800 m/z. MS/MS spectra were acquired in data-dependent acquisition mode at top speed for 3 s per cycle; the AGC target was set to 1E4 with maximum injection time of 35 ms. Ions were subjected to stepped-energy higher-energy collision dissociation (seHCD) fragmentation at a normalized collision energy (NCE) of 20±5%.

Label-free quantification analysis

The raw LC-MS/MS data files were analyzed using MaxQuant (version 2.0.3.1), with the spectra searched against the wild-type and mutant rhodopsin sequences. For identification of the peptides, the mass tolerances were 20 ppm for initial precursor ions, and 0.5 Da for fragment ions. Two missed cleavages in tryptic digests were allowed. Cysteine residues were set as static modifications. Oxidation of methionine was set as the variable modification. Filtering for the peptide identification was set at a 1% false discovery rate (FDR).

CIRCLE-seq off-target analysis

CIRCLE-seq off-target editing analysis was performed as previously described (3, 4). Genomic DNA from Rho-E150K mouse liver was isolated using Gentra Puregene Kit (Qiagen #158845), following the manufacturer's instructions. Purified genomic DNA was sheared with a Covaris S2 instrument to an average length of 300 bp. The fragmented DNA was end-repaired, A-tailed and ligated to a uracil-containing stem–loop adaptor, using the KAPA HTP Library Preparation Kit, PCR Free (KAPA Biosystems #KK8235). Adaptor-ligated DNA was treated with Lambda Exonuclease (New England Biolabs #M0262) and *E. coli* Exonuclease I (New England Biolabs #M0293); and then with USER enzyme (New England Biolabs #M5505) and T4 poly-nucleotide kinase (New England Biolabs #M0201). Intramolecular circularization of the DNA was performed with T4 DNA ligase (New England Biolabs #M0202), and residual linear DNA was degraded by Plasmid-Safe ATP-dependent DNase (Lucigen #E3110). Synthetic guide RNAs were ordered from IDT with standard 2′-*O*-methyl modification at the first three and last three bases. The synthetic guide RNAs were resuspended to 9 µM in nuclease-free water, denatured at 90 °C for 5 min and slowly annealed while cooling at 0.1 °C s^{-1} to 25 °C. *In vitro* cleavage reactions were performed with 125 ng Plasmid-Safe-treated circularized DNA, 90 nM Cas9 nuclease protein (New England Biolabs #M0386) and 270 nM synthetic guide RNA in a 50 µl volume for 1 h. Cleaved products were treated with proteinase K as described, A-tailed, ligated with a hairpin adaptor (New England Biolabs #E7600S), treated with USER enzyme (New England Biolabs #M5505), and amplified by PCR with barcoded universal primers (New England Biolabs #E7600S), using Kapa HiFi Polymerase (KAPA Biosystems #KK4824). Libraries were sequenced with 150-bp/150-bp paired-end reads with an Illumina MiSeq instrument. CIRCLE-seq data analyses were performed using open-source CIRCLE-seq analysis software and recommended default parameters. The top ten nominated off-target sites for the Rho-E150K sgRNA were analyzed by HTS from the retinas of untreated or dual-AAV8 treated mice. Insertions or deletions at sgRNA-associated off-target sites were analyzed as a percentage of discarded reads divided by the total reference-aligned reads. All CIRCLE-seq nominated off-target sites are listed in **Supplementary Table 4**.

High-throughput sequencing data analysis

Sequencing reads were demultiplexed using the MiSeq Reporter software (Illumina), and were analyzed using CRISPResso2 (5). Batch analysis mode (one batch for each unique amplicon and sgRNA combination analyzed) was used in all cases. Reads were filtered according to minimum average quality score (Q > 30) prior to analysis. The following quantification window parameters

were used: -w 20, -wc -10. Base-editing efficiencies are reported as the percentage of sequencing reads containing a given base conversion at a specific position.

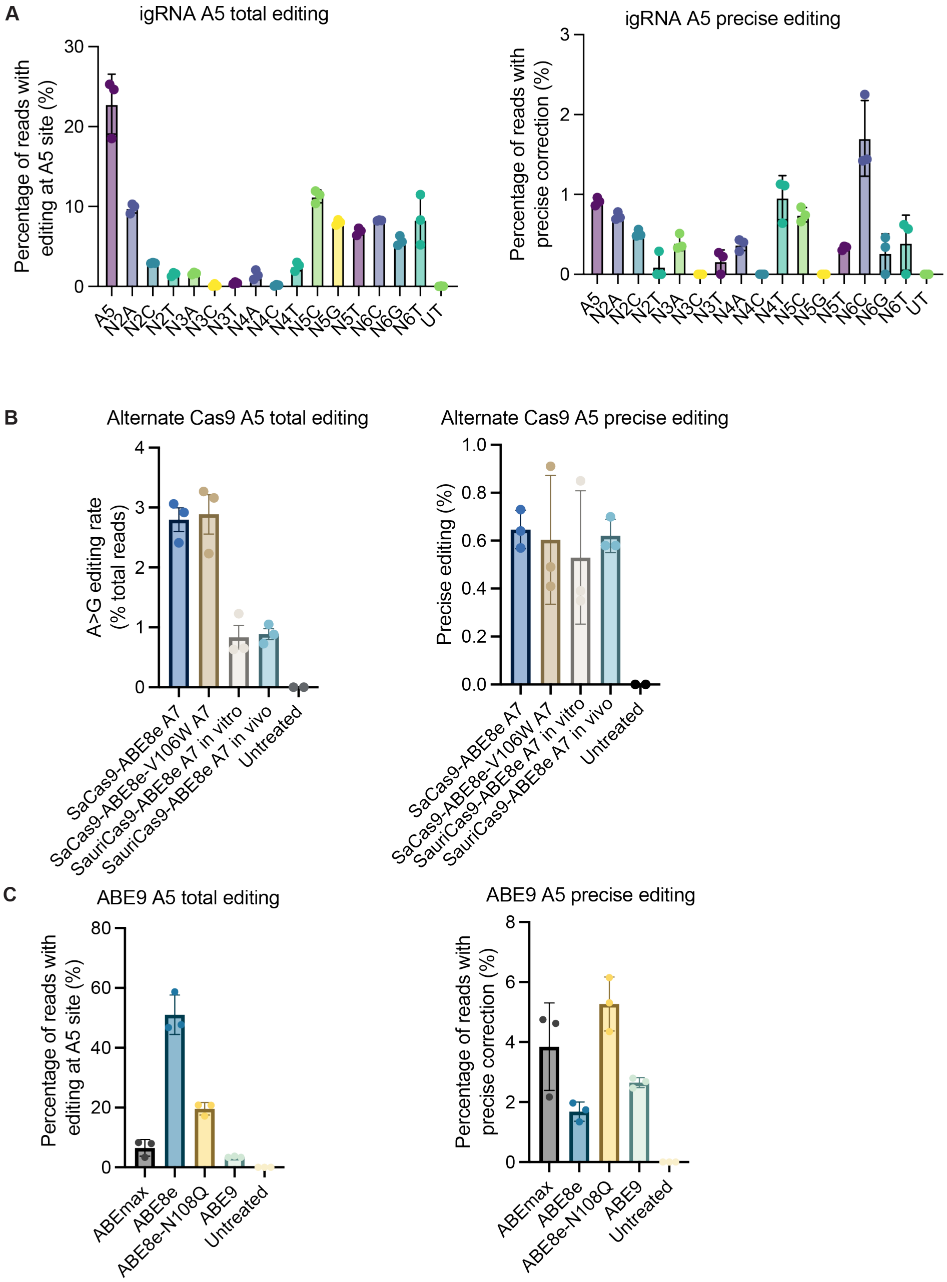

Supplemental Figure 1. Screening of alternate base-editing strategies *in vitro*. (**A**) NGS results of imperfect guide RNA (igRNA) plus ABE8e, screening with total-target base editing (left) and precise-target base editing (right). igRNAs are denoted by position in the protospacer (N) and the mutated base. UT, untreated. (**B**) NGS results of alternative Cas9-ABEs, screening with total-target base editing (left) and precise-target base editing (right). (**C**) NGS results of ABE9-variant screening with total-target base editing (left) and precise-target base editing (right). All results are represented as mean ± S.D.

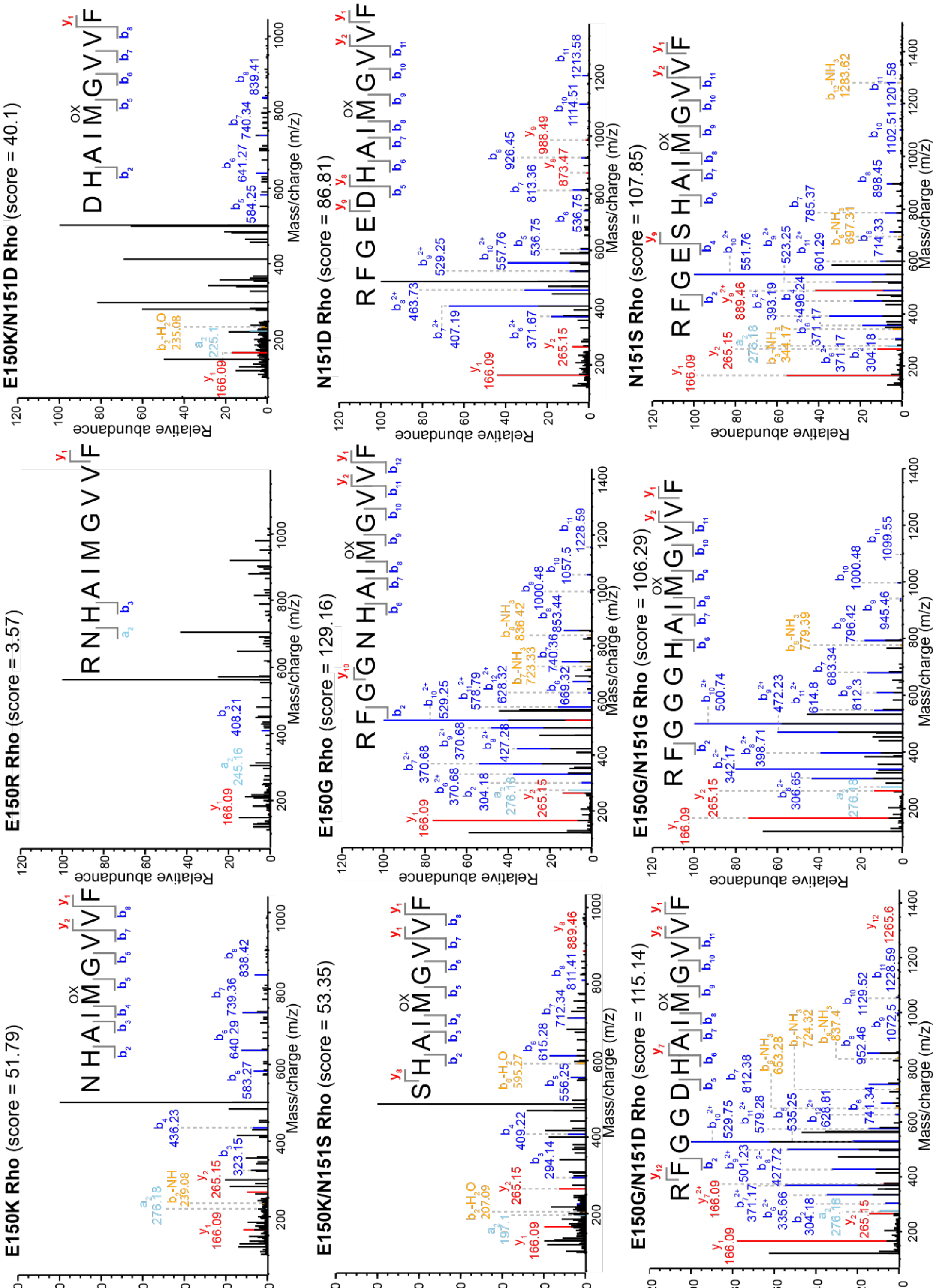

Supplemental Figure 2. Tandem MS/MS spectra of purified peptides from rhodopsin variants. Spectra for each unique rhodopsin variant and their respective peptide fragmentation patterns.

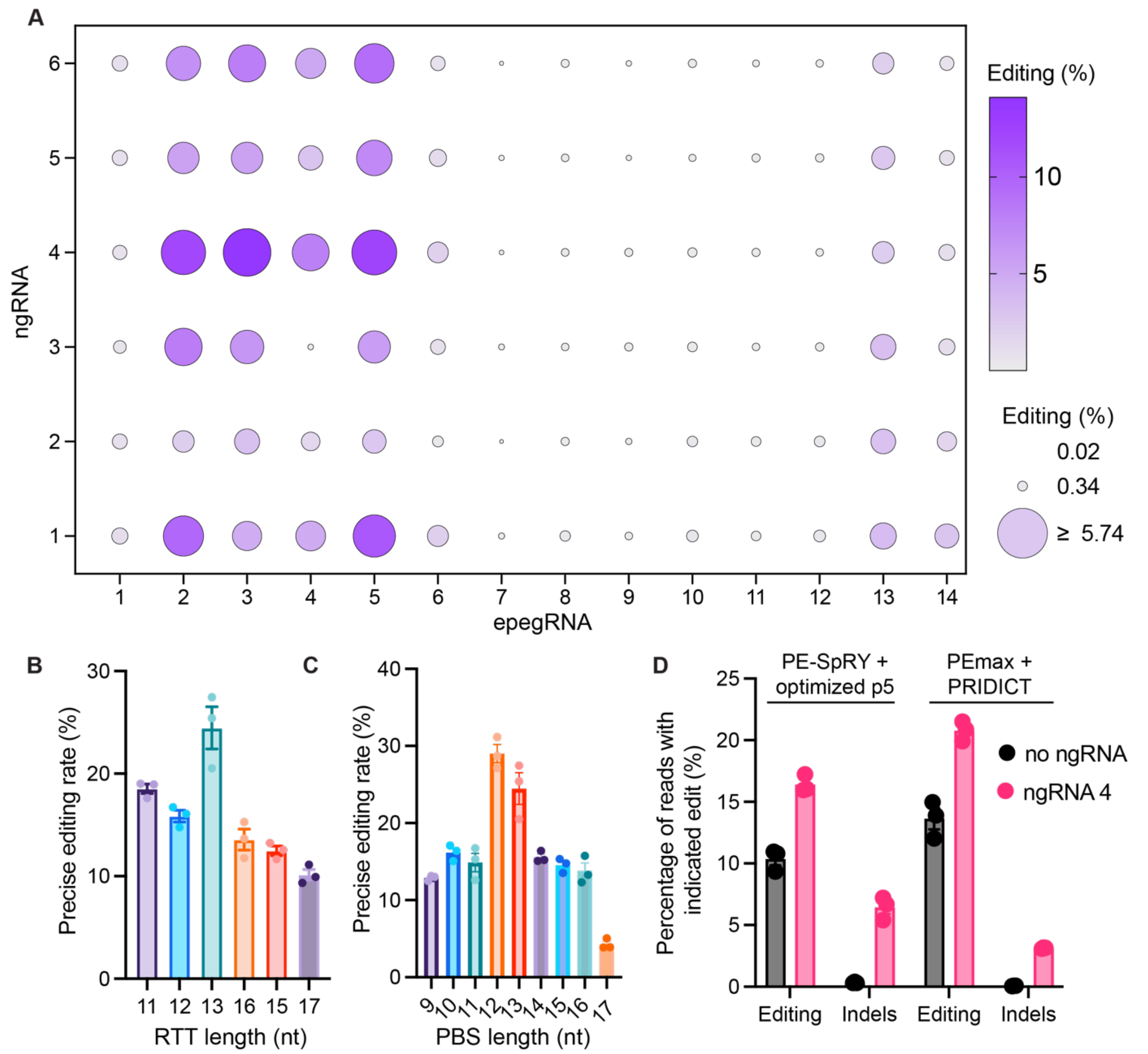

Supplemental Figure 3. *In vitro* screening of engineered prime-editing guide RNAs (epegRNAs) for correction of Rho-E150K. (**A**) Bubble plot of epegRNA and nicking guide RNA (ngRNA) screening for PE-SpRY. Bubbles are colored and sized according to precise A>G editing rate. (**B, C**) Optimization of lengths of p5 epegRNA reverse-transcriptase template (RTT, left) and primer binding site (PBS, middle). (**D**) Comparison of PE-SpRY and optimized p5 epegRNA with PEmax and PRIDICT epegRNA. Results are represented as mean ± S.D.

Supplemental Table 1. PCR primers used in this study.

Primer name	Sequence	Purpose
gDNA-NGS-F	ACACTCTTTCCCTACACGACGCTCTTCCGATCTNNNNGCCCAAACTCACTGCAGTCGCT	Amplification of genomic DNA
gDNA-NGS-R	TGGAGTTCAGACGTGTGCTCTTCCGATCTGCCAGACCCGATACTCAGTGCC	Amplification of genomic DNA
cDNA-NGS-F	ACACTCTTTCCCTACACGACGCTCTTCCGATCTNNNNGGTCCTGGCCATTGAGCGCTAC	Amplification of complementary DNA, cell line
cDNA-NGS-R	TGGAGTTCAGACGTGTGCTCTTCCGATCTTGACTGTGAAGACCAGCTGCCCA	Amplification of complementary DNA, cell line
mRho-InFusion-F	GTGGAATTCGCCACCATGAACGGCACCGAGGG	InFusion cloning of mouse rhodopsin from cDNA
mRho-InFusion-R	TAGACTCGAGCGGCCTCAGGCAGGGGCCACCTGGC	InFusion cloning of mouse rhodopsin from cDNA
Q5SDM_A6_F	CGCTTCGGGagGAATCACGCT	Forward primer for mouse Rho mutagenization
Q5SDM_A8_F	CGCTTCGGGaAGgATCACGCTAT	Forward primer for mouse Rho mutagenization
Q5SDM_A5+A6_F	CGCTTCGGGGgGAATCACGCT	Forward primer for mouse Rho mutagenization

Q5SDM_A5 +A8_F	CGCTTCGGGGAGgATCACGCTATC	Forward primer for mouse Rho mutagenization
Q5SDM_A5 +A6+A8_F	CGCTTCGGGGgGgATCACGCTATC	Forward primer for mouse Rho mutagenization
Q5SDM_A5 +A6+A8+A9 _F	CGCTTCGGGGgGggTCACGCTATC	Forward primer for mouse Rho mutagenization
Q5SDM_A5 +A9_F	CGCTTCGGGGAGAgTCACGCTATC	Forward primer for mouse Rho mutagenization
Q5SDM_A9 _F	CGCTTCGGGaAGAgTCACGCTATC	Forward primer for mouse Rho mutagenization
Q5SDM_A5 _F	CGCTTCGGGgaGAATCACGCT	Forward primer for mouse Rho mutagenization
Q5SDM_R	GAAGTTGCTCATCGGCTTGCAG	Reverse primer for mouse Rho mutagenization
OT1-EK-F	ACACTCTTTCCCTACACGACGCTCTTCCGATCTNNNNAC ATGGCATCTGCTATTGTCCT	CIRCLE-seq off-target
OT1-EK-R	TGGAGTTCAGACGTGTGCTCTTCCGATGCCGGCTTTCGA TTTCTCAG	CIRCLE-seq off-target
OT2-EK-F	ACACTCTTTCCCTACACGACGCTCTTCCGATCTNNNNCA GGCTAATTTGCTAGAGGTCA	CIRCLE-seq off-target
OT2-EK-R	TGGAGTTCAGACGTGTGCTCTTCCGATGCAGCAAAGTTG GTAGGTGG	CIRCLE-seq off-target
OT3-EK-F	ACACTCTTTCCCTACACGACGCTCTTCCGATCTNNNNCA GAGGTGGATGCTCACAGC	CIRCLE-seq off-target
OT3-EK-R	TGGAGTTCAGACGTGTGCTCTTCCGATCCCCGTTCTCAA ATTCCCCT	CIRCLE-seq off-target

OT4-EK-F	ACACTCTTTCCCTACACGACGCTCTTCCGATCTNNNNNCC AGAGGTAGCTCTCCCCAT	CIRCLE-seq off-target
OT4-EK-R	TGGAGTTCAGACGTGTGCTCTTCCGATTCACTCTCTCAA AACATATCACCCA	CIRCLE-seq off-target
OT5-EK-F	ACACTCTTTCCCTACACGACGCTCTTCCGATCTNNNNNGA AGTTCTGCCAGAGCCTGA	CIRCLE-seq off-target
OT5-EK-R	TGGAGTTCAGACGTGTGCTCTTCCGATTCCCCTACACTG GAGCATCA	CIRCLE-seq off-target
OT6-EK-F	ACACTCTTTCCCTACACGACGCTCTTCCGATCTNNNNNAG GCAGCCTGGTTTCTATGT	CIRCLE-seq off-target
OT6-EK-R	TGGAGTTCAGACGTGTGCTCTTCCGATTGATGGTGACAC CCTTGCTC	CIRCLE-seq off-target
OT7-EK-F	ACACTCTTTCCCTACACGACGCTCTTCCGATCTNNNNNAG GCCTTGCGTGTCAGATAG	CIRCLE-seq off-target
OT7-EK-R	TGGAGTTCAGACGTGTGCTCTTCCGATCATGTTTGTATG TCCCATCTGCT	CIRCLE-seq off-target
OT8-EK-F	ACACTCTTTCCCTACACGACGCTCTTCCGATCTNNNNNCT CAGGGCTGTTGGTCTTGT	CIRCLE-seq off-target
OT8-EK-R	TGGAGTTCAGACGTGTGCTCTTCCGATGCCATGTCCCCA TCAGTTCA	CIRCLE-seq off-target
OT9-EK-F	ACACTCTTTCCCTACACGACGCTCTTCCGATCTNNNNNAC TGGAACTTCATGGGCAAAAG	CIRCLE-seq off-target
OT9-EK-R	TGGAGTTCAGACGTGTGCTCTTCCGATCTGGAGAGGGC CGACATCA	CIRCLE-seq off-target
OT10-EK-F	ACACTCTTTCCCTACACGACGCTCTTCCGATCTNNNNNCA TGTCCGCACTTACCTCCTAA	CIRCLE-seq off-target
OT10-EK-R	TGGAGTTCAGACGTGTGCTCTTCCGATGGAAAAGTTCCT CTCGGTCCT	CIRCLE-seq off-target
A5-KLD-F	GCGGGaAGAATCACGCTATCAGTTTTAGAGCTAGAA ATAGCAAGTTAAAATAAG	KLD forward primer for sgRNA insertion

A6-KLD-F	GTCGGGaAGAATCACGCTATCGTTTTAGAGCTAGAAATAGCAAGTTAAAATAAG	KLD forward primer for sgRNA insertion
A7-KLD-F	GTTCGGGaAGAATCACGCTATGTTTTAGAGCTAGAAATAGCAAGTTAAAATAAG	KLD forward primer for sgRNA insertion
A9-KLD-F	GCTTCGGGaAGAATCACGCTGTTTTAGAGCTAGAAATAGCAAGTTAAAATAAG	KLD forward primer for sgRNA insertion
A10-KLD-F	GCGCTTCGGGaAGAATCACGCGTTTTAGAGCTAGAAATAGCAAGTTAAAATAAG	KLD forward primer for sgRNA insertion
A11-KLD-F	GCCGCTTCGGGaAGAATCACGGTTTTAGAGCTAGAAATAGCAAGTTAAAATAAG	KLD forward primer for sgRNA insertion
KLD-R	GGTGTTTCGTCCTTTCCACAAG	KLD reverse primer for sgRNA insertion
r11	CGG GGA GAA TCG CAC CGA CTC G	KLD mutagenesis of p5 RTT
r12	CGG GGA GAA TCA GCA CCG ACT CG	KLD mutagenesis of p5 RTT
r15	CGG GGA GAA TCA CGC GCA CCG ACT CG	KLD mutagenesis of p5 RTT

r16	CGG GGA GAA TCA CGC TGC ACC GAC TCG	KLD mutagenesis of p5 RTT
r17	CGG GGA GAA TCA CGC TAG CAC CGA CTC G	KLD mutagenesis of p5 RTT
p9	AAG CGG AAG TTG CCG CGG TTC TAT CTA	KLD mutagenesis of p5 PBS
p10	AAG CGG AAG TCG CGG TTC TAT CTA	KLD mutagenesis of p5 PBS
p11	AAG CGG AAG TTC GCG GTT CTA TCT A	KLD mutagenesis of p5 PBS
p12	AAG CGG AAG TTG CGC GGT TCT ATC TA	KLD mutagenesis of p5 PBS
p14	AAG CGG AAG TTG CGC GCG GTT CTA TCT A	KLD mutagenesis of p5 PBS
p15	AAG CGG AAG TTG CGG CGC GGT TCT ATC TA	KLD mutagenesis of p5 PBS
p16	AAG CGG AAG TTG CGG ACG CGG TTC TAT CTA	KLD mutagenesis of p5 PBS
p17	AAG CGG AAG TTG CGG AAC GCG GTT CTA TCT A	KLD mutagenesis of p5 PBS
r-p13F	AAG CGG AAG TTG CCG CGG TTC TAT CTA	Reverse primer for KLD

		mutagenesis of p5 RTT
p-r13r	CGG GGA GAA TCA CGC ACC GAC TCG	Reverse primer for KLD mutagenesis of p5 PBS

Supplemental Table 2. Key plasmid sequences used in this study.

>pAAV.Cbh.Nterm.ABEmax, N-terminal AAV ABEmax, Addgene #137177

ccactccctctctgcgcgctcgctcgctcactgaggccgggcgaccaaaggtcgcccgacgcccgggctttgcccgggcggcctca
gtgagcgagcgagcgcgcagccttaattaacctaattcactggccgtcgttttacaacgtcgtgactgggaaaaccctggcgttaccca
acttaatcgccttgcagcacatccccctttcgccagctggcgtaatagcgaagaggcccgcaccgatcgcccttcccaacagttgcgc
agcctgaatggcgaatgggacgcgccctgtagcggcgcattaagcgcggcgggtgtggtggttacgcgcagcgtgaccgctacact
tgccagcgccctagcgcccgctcctttcgctttcttcccttcctttctcgccacgttcgccggctttccccgtcaagctctaaatcgggggctc
cctttagggttccgatttagtgctttacggcacctcgaccccaaaaaacttgattagggtgatggttcacgtagtgggccatcgccctgat
agacggtttttcgccctttgacgttggagtccacgttctttaatagtggactcttgttccaaactggaacaacactcaaccctatctcggtcta
ttcttttgatttataagggattttgccgatttcggcctattggttaaaaaatgagctgatttaacaaaaatttaacgcgaattttaacaaaatatt
aacgtttataatttcaggtggcatctttcggggaaatgtgcgcggaacccctatttgtttatttttctaaatacattcaaatatgtatccgctcat
gagacaataaccctgataaatgcttcaataatattgaaaaaggaagagtatgagtattcaacatttccgtgtcgcccttattcccttttttgc
ggcattttgccttcctgtttttgctcacccagaaacgctggtgaaagtaaaagatgctgaagatcagttgggtgcacgagtgggttacatc
gaactggatctcaacagcggtaagatccttgagagttttcgccccgaagaacgttttccaatgatgagcacttttaaagttctgctatgtg
gcgcggtattatcccgtattgacgccgggcaagagcaactcggtcgccgcatacactattctcagaatgacttggttgagtactcacca
gtcacagaaaagcatcttacggatggcatgacagtaagagaattatgcagtgctgccataaccatgagtgataacactgcggccaa
cttacttctgacaacgatcggaggaccgaaggagctaaccgcttttttgcacaacatgggggatcatgtaactcgccttgatcgttgga
accggagctgaatgaagccataccaaacgacgagcgtgacaccacgatgcctgtagtaatggtaacaacgttgcgcaaactattaa
ctggcgaactacttactctagcttcccggcaacaattaatagactggatggaggcggataaagttgcaggaccacttctgcgctcggc
ccttccggctggctggtttattgctgataaatctggagccggtgagcgtgggtctcgcggtatcattgcagcactggggccagatggtaa
gccctcccgtatcgtagttatctacacgacggggagtcaggcaactatggatgaacgaaatagacagatcgctgagataggtgcctc
actgattaagcattggtaactgtcagaccaagtttactcatatatactttagattgatttaaaacttcatttttaatttaaaaggatctaggtga
agatcctttttgataatctcatgaccaaaatcccttaacgtgagttttcgttccactgagcgtcagaccccgtagaaaagatcaaaggatc
ttcttgagatcctttttttctgcgcgtaatctgctgcttgcaaacaaaaaaaccaccgctaccagcggtggtttgtttgccggatcaagagct
accaactctttttccgaaggtaactggcttcagcagagcgcagataccaaatactgtccttctagtgtagccgtagttaggccaccacttc
aagaactctgtagcaccgcctacatacctcgctctgctaatcctgttaccagtggctgctgccagtggcgataagtcgtgtcttaccgggt
tggactcaagacgatagttaccggataaggcgcagcggtcgggctgaacggggggttcgtgcacacagcccagcttggagcgaac
gacctacaccgaactgagatacctacagcgtgagctatgagaaagcgccacgcttcccgaagggagaaaggcggacaggtatcc
ggtaagcggcagggtcggaacaggagagcgcacgagggagcttccagggggaaacgcctggtatctttatagtcctgtcgggtttc
gccacctctgacttgagcgtcgatttttgtgatgctcgtcaggggggcggagcctatggaaaaacgccagcaacgcggcctttttacgg
ttcctggccttttgctgcggttttgctcacatgttctttcctgcgttatcccctgattctgtggataaccgtattaccgcctttgagtgagctgatac
cgctcgccgcagccgaacgaccgagcgcagcgagtcagtgagcgaggaagcggaagagcgcccaatacgcaaaccgcctctc
cccgcgcgttggccgattcattaatgcagctggcacgacaggtttcccgactggaaagcgggcagtgagcgcaacgcaattaatgtg
agttagctcactcattaggcaccccaggctttacactttatgcttccggctcgtatgttgtgtggaattgtgagcggataacaatttcacaca

ggaaacagctatgaccatgattacgccagatttaattaaggctgcgcgctcgctcgctcactgaggccgcccgggcaaagcccggg
cgtcgggcgacctttggtcgcccggcctcagtgagcgagcgagcgcgcagagagggagtggccaactccatcactaggggttcctg
cggcctctagatcagggtacccgttacataacttacggtaaatggcccgcctggctgaccgcccaacgacccccgcccattgacgtc
aatagtaacgccaatagggactttccattgacgtcaatgggtggagtatttacggtaaactgcccacttggcagtacatcaagtgtatca
tatgccaagtacgccccctattgacgtcaatgacggtaaatggcccgcctggcattgtgcccagtacatgaccttatgggactttcctact
tggcagtacatctacgtattagtcatcgctattaccatggtcgaggtgagccccacgttctgcttcactctccccatctcccccccctcccc
acccccaattttgtatttatttattttttaattattttgtgcagcgatgggggcggggggggggggggcgcgcgccaggcggggcggg
gcggggcgaggggcggggcggggcgaggcggagaggtgcggcggcagccaatcagagcggcgcgctccgaaagtttccttttat
ggcgaggcggcggcggcggcggccctataaaaagcgaagcgcgcggcgggcgggagtcgctgcgacgctgccttcgccccgtg
ccccgctccgccgccgcctcgcgccgcccgccccggctctgactgaccgcgttactcccacaggtgagcgggcgggacggcccttc
tcctccgggctgtaattagctgagcaagaggtaagggtttaagggatggttggttggtggggtattaatgtttaattacctggagcacctg
cctgaaatcactttttttcaggttggaccggtgccaccatgaaacggacagccgacggaagcgagttcgagtcaccaaagaagaag
cggaaagtctctgaagtcgagtttagccacgagtattggatgaggcacgcactgaccctggcaaagcgagcatgggatgaaagag
aagtccccgtgggcgccgtgctggtgcacaacaatagagtgatcggagagggatggaacaggccaatcggccgccacgacccta
ccgcacacgcagagatcatggcactgaggcagggaggcctggtcatgcagaattaccgcctgatcgatgccaccctgtatgtgaca
ctggagccatgcgtgatgtgcgcaggagcaatgatccacagcaggatcggaagagtggtgttcggagcacgggacgccaagacc
ggcgcagcaggctccctgatggatgtgctgcaccaccccggcatgaaccaccgggtggagatcacagagggaatcctggcagac
gagtgcgccgccctgctgagcgatttctttagaatgcggagacaggagatcaaggcccagaagaaggcacagagctccaccgact
ctggaggatctagcggaggatcctctggaagcgagacaccaggcacaagcgagtccgccacaccagagagctccggcggctcct
ccggaggatcctctgaggtggagttttcccacgagtactggatgagacatgccctgaccctggccaagagggcacgcgatgagagg
gaggtgcctgtgggagccgtgctggtgctgaacaatagagtgatcggcgagggctggaacagagccatcggcctgcacgacccaa
cagcccatgccgaaattatggccctgagacagggcggcctggtcatgcagaactacagactgattgacgccaccctgtacgtgacat
tcgagccttgcgtgatgtgcgccggcgccatgatccactctaggatcggccgcgtggtgtttggcgtgaggaacgcaaaaaccggcg
ccgcaggctccctgatggacgtgctgcactaccccggcatgaatcaccgcgtcgaaattaccgagggaatcctggcagatgaatgtg
ccgccctgctgtgctatttctttcggatgcctagacaggtgttcaatgctcagaagaaggcccagagctccaccgactccggaggatct
agcggaggctcctctggctctgagacacctggcacaagcgagagcgcaacacctgaaagcagcgggggcagcagcgggggt
cagacaagaagtacagcatcggcctggccatcggcaccaactctgtgggctgggccgtgatcaccgacgagtacaaggtgcccag
caagaaattcaaggtgctgggcaacaccgaccggcacagcatcaagaagaacctgatcggagccctgctgttcgacagcggcga
aacagccgaggccacccggctgaagagaaccgccagaagaagatacaccagacggaagaaccggatctgctatctgcaagag
atcttcagcaacgagatggccaaggtggacgacagcttcttccacagactggaagagtccttcctggtggaagaggataagaagca
cgagcggcaccccatcttcggcaacatcgtggacgaggtggcctaccacgagaagtaccccaccatctaccacctgagaaagaa
actggtggacagcaccgacaaggccgacctgcggctgatctatctggccctggcccacatgatcaagttccggggccacttcctgatc
gagggcgacctgaaccccgacaacagcgacgtggacaagctgttcatccagctggtgcagacctacaaccagctgttcgaggaaa
accccatcaacgccagcggcgtggacgccaaggccatcctgtctgccagactgagcaagagcagacggctggaaaatctgatcg

cccagctgcccggcgagaagaagaatggcctgttcggaaacctgattgccctgagcctgggcctgacccccaacttcaagagcaa
cttcgacctggccgaggatgccaaactgcagctgagcaaggacacctacgacgacgacctggacaacctgctggcccagatcgg
cgaccagtacgccgacctgtttctggccgccaagaacctgtccgacgccatcctgctgagcgacatcctgagagtgaacaccgagat
caccaaggcccccctgagcgcctctatgatcaagagatacgacgagcaccaccaggacctgaccctgctgaaagctctcgtgcgg
cagcagctgcctgagaagtacaaagagattttcttcgaccagagcaagaacggctacgccggctacattgacggcggagccagcc
aggaagagttctacaagttcatcaagcccatcctggaaaagatggacggcaccgaggaactgctcgtgaagctgaacagagagg
acctgctgcggaagcagcggaccttcgacaacggcagcatcccccaccagatccacctgggagagctgcacgccattctgcggcg
gcaggaagatttttacccattcctgaaggacaaccgggaaaagatcgagaagatcctgaccttccgcatcccctactacgtgggccct
ctggccaggggaaacagcagattcgcctggatgaccagaaagagcgaggaaaccatcacccccctggaacttcgaggaagtggtg
gacaagggcgcttccgcccagagcttcatcgagcggatgaccaacttcgataagaacctgcccaacgagaaggtgctgcccaagc
acagcctgctgtacgagtacttcaccgtgtataacgagctgaccaaagtgaaatacgtgaccgagggaatgagaaagcccgccttc
ctgagcggcgagcagaaaaaggccatcgtggacctgctgttcaagaccaaccggaaagtgaccgtgaagcagctgaaagagga
ctacttcaagaaaatcgagtgcctgtcctacgagacagagatcctgacagtggagtatggcctgctgccaatcggcaagatcgtgga
gaagaggatcgagtgtaccgtgtactctgtggataacaatggcaacatctatacacagcccgtggcacagtggcacgataggggag
agcaggaggtgttcgagtattgcctggaggacggcagcctgatcagggcaaccaaggaccacaagttcatgacagtggatggcca
gatgctgcccatcgacgagattttcgagcgggagctggacctgatgagagtggataacctgcctaatagcggaggcagtaaaagaa
cagcagacgggagtgagtttgagcccaagaaaaagagaaaggtgtaagatctgataatcaacctctggattacaaaatttgtgaaa
gattgactggtattcttaactatgttgctccttttacgctatgtggatacgctgctttaatgcctttgtatcatgctattgcttcccgtatggctttcat
tttctcctccttgtataaatcctggttagttcttgccacggcggaactcatcgccgcctgccttgcccgctgctggacaggggctcggctgtt
gggcactgacaattccgtggtgcgactgtgccttctagttgccagccatctgttgtttgcccctcccccgtgccttccttgaccctggaaggt
gccactcccactgtcctttcctaataaaatgaggaaattgcatcgcattgtctgagtaggtgtcattctattctggggggtggggtggggc
aggacagcaagggggaggattgggaagacaatagcaggcatgctggggatgcggtgggctctatgggcggccgcaggaacccc
tagtgatggagttgg

>pAAV.Cbh.Cterm.ABEmax.E150K.A5, C-terminal AAV ABEmax with A5 E150K sgRNA, derived from Addgene #137179
ccactccctctctgcgcgctcgctcgctcactgaggccgggcgaccaaaggtcgcccgacgcccgggctttgcccgggcggcctca
gtgagcgagcgagcgcgcagccttaattaacctaattcactggccgtcgttttacaacgtcgtgactgggaaaaccctggcgttaccca
acttaatcgccttgcagcacatccccctttcgccagctggcgtaatagcgaagaggcccgcaccgatcgcccttcccaacagttgcgc
agcctgaatggcgaatggacgcgccctgtagcggcgcattaagcgcggcgggtgtggtggttacgcgcagcgtgaccgctacact
tgccagcgccctagcgcccgctcctttcgctttcttcccttcctttctcgccacgttcgccggctttccccgtcaagctctaaatcgggggctc
cctttagggttccgatttagtgctttacggcacctcgaccccaaaaaacttgattagggtgatggttcacgtagtgggccatcgccctgat
agacggtttttcgccctttgacgttggagtccacgttctttaatagtggactcttgttccaaactggaacaacactcaaccctatctcggtcta
ttctttgatttataagggattttgccgatttcggcctattggttaaaaaatgagctgatttaacaaaaatttaacgcgaattttaacaaaatatt

aacgtttataatttcaggtggcatctttcggggaaatgtgcgcggaacccctatttgtttatttttctaaatacattcaaatatgtatccgctcat
gagacaataaccctgataaatgcttcaataatattgaaaaaggaagagtatgagtattcaacatttccgtgtcgcccttattcccttttttgc
ggcattttgccttcctgtttttgctcacccagaaacgctggtgaaagtaaaagatgctgaagatcagttgggtgcacgagtgggttacatc
gaactggatctcaacagcggtaagatccttgagagttttcgccccgaagaacgttttccaatgatgagcacttttaaagttctgctatgtg
gcgcggtattatcccgtattgacgccgggcaagagcaactcggtcgccgcatacactattctcagaatgacttggttgagtactcacca
gtcacagaaaagcatcttacggatggcatgacagtaagagaattatgcagtgctgccataaccatgagtgataacactgcggccaa
cttacttctgacaacgatcggaggaccgaaggagctaaccgcttttttgcacaacatgggggatcatgtaactcgccttgatcgttgga
accggagctgaatgaagccataccaaacgacgagcgtgacaccacgatgcctgtagtaatggtaacaacgttgcgcaaactattaa
ctggcgaactacttactctagcttcccggcaacaattaatagactggatggaggcggataaagttgcaggaccacttctgcgctcggc
ccttccggctggctggtttattgctgataaatctggagccggtgagcgtgggtctcgcggtatcattgcagcactggggccagatggtaa
gccctcccgtatcgtagttatctacacgacggggagtcaggcaactatggatgaacgaaatagacagatcgctgagataggtgcctc
actgattaagcattggtaactgtcagaccaagtttactcatatatactttagattgatttaaaacttcatttttaatttaaaaggatctaggtga
agatcctttttgataatctcatgaccaaaatcccttaacgtgagttttcgttccactgagcgtcagaccccgtagaaaagatcaaaggatc
ttcttgagatcctttttttctgcgcgtaatctgctgcttgcaaacaaaaaaaccaccgctaccagcggtggtttgtttgccggatcaagagct
accaactctttttccgaaggtaactggcttcagcagagcgcagataccaaatactgtccttctagtgtagccgtagttaggccaccacttc
aagaactctgtagcaccgcctacatacctcgctctgctaatcctgttaccagtggctgctgccagtggcgataagtcgtgtcttaccgggt
tggactcaagacgatagttaccggataaggcgcagcggtcgggctgaacggggggttcgtgcacacagcccagcttggagcgaac
gacctacaccgaactgagatacctacagcgtgagctatgagaaagcgccacgcttcccgaagggagaaaggcggacaggtatcc
ggtaagcggcagggtcggaacaggagagcgcacgagggagcttccagggggaaacgcctggtatctttatagtcctgtcgggtttc
gccacctctgacttgagcgtcgatttttgtgatgctcgtcaggggggcggagcctatggaaaaacgccagcaacgcggcctttttacgg
ttcctggccttttgctgcggttttgctcacatgttctttcctgcgttatcccctgattctgtggataaccgtattaccgcctttgagtgagctgatac
cgctcgccgcagccgaacgaccgagcgcagcgagtcagtgagcgaggaagcggaagagcgcccaatacgcaaaccgcctctc
cccgcgcgttggccgattcattaatgcagctggcacgacaggtttcccgactggaaagcgggcagtgagcgcaacgcaattaatgtg
agttagctcactcattaggcaccccaggctttacactttatgcttccggctcgtatgttgtgtggaattgtgagcggataacaatttcacaca
ggaaacagctatgaccatgattacgccagatttaattaaggctgcgcgctcgctcgctcactgaggccgcccgggcaaagcccggg
cgtcgggcgacctttggtcgcccggcctcagtgagcgagcgagcgcgcagagagggagtggccaactccatcactaggggttcctg
cggcctctagatcagggtacccgttacataacttacggtaaatggcccgcctggctgaccgcccaacgacccccgcccattgacgtc
aatagtaacgccaatagggactttccattgacgtcaatgggtggagtatttacggtaaactgcccacttggcagtacatcaagtgtatca
tatgccaagtacgccccctattgacgtcaatgacggtaaatggcccgcctggcattgtgcccagtacatgaccttatgggactttcctact
tggcagtacatctacgtattagtcatcgctattaccatggtcgaggtgagccccacgttctgcttcactctccccatctcccccccctcccc
acccccaattttgtatttatttattttttaattattttgtgcagcgatgggggcgggggggggggggggcgcgcgccaggcggggcggg
gcggggcgaggggcggggcggggcgaggcggagaggtgcggcggcagccaatcagagcggcgcgctccgaaagtttccttttat
ggcgaggcggcggcggcggcggccctataaaaagcgaagcgcgcggcgggcgggagtcgctgcgacgctgccttcgccccgtg
ccccgctccgccgccgcctcgcgccgcccgccccggctctgactgaccgcgttactcccacaggtgagcgggcgggacggcccttc

tcctccgggctgtaattagctgagcaagaggtaagggtttaagggatggttggttggtggggtattaatgtttaattacctggagcacctg
cctgaaatcactttttttcaggttggaccggtgccaccatgaaacggacagccgacggaagcgagttcgagtcaccaaagaagaag
cggaaagtcatcaagattgctacacggaaatacctgggaaagcagaacgtgtacgacatcggcgtggagcgggatcacaacttcg
ccctgaagaatggctttatcgccagcaattgcttcgactccgtggaaatctccggcgtggaagatcggttcaacgcctccctgggcaca
taccacgatctgctgaaaattatcaaggacaaggacttcctggacaatgaggaaaacgaggacattctggaagatatcgtgctgacc
ctgacactgtttgaggacagagagatgatcgaggaacggctgaaaacctatgcccacctgttcgacgacaaagtgatgaagcagct
gaagcggcggagatacaccggctggggcaggctgagccggaagctgatcaacggcatccgggacaagcagtccggcaagaca
atcctggatttcctgaagtccgacggcttcgccaacagaaacttcatgcagctgatccacgacgacagcctgacctttaaagaggaca
tccagaaagcccaggtgtccggccagggcgatagcctgcacgagcacattgccaatctggccggcagccccgccattaagaagg
gcatcctgcagacagtgaaggtggtggacgagctcgtgaaagtgatgggccggcacaagcccgagaacatcgtgatcgaaatgg
ccagagagaaccagaccacccagaagggacagaagaacagccgcgagagaatgaagcggatcgaagagggcatcaaaga
gctgggcagccagatcctgaaagaacaccccgtggaaaacacccagctgcagaacgagaagctgtacctgtactacctgcagaat
gggcgggatatgtacgtggaccaggaactggacatcaaccggctgtccgactacgatgtggaccatatcgtgcctcagagctttctga
aggacgactccatcgacaacaaggtgctgaccagaagcgacaagaaccggggcaagagcgacaacgtgccctccgaagaggt
cgtgaagaagatgaagaactactggcggcagctgctgaacgccaagctgattacccagagaaagttcgacaatctgaccaaggcc
gagagaggcggcctgagcgaactggataaggccggcttcatcaagagacagctggtggaaacccggcagatcacaaagcacgt
ggcacagatcctggactcccggatgaacactaagtacgacgagaatgacaagctgatccgggaagtgaaagtgatcaccctgaag
tccaagctggtgtccgatttccggaaggatttccagttttacaaagtgcgcgagatcaacaactaccaccacgcccacgacgcctacc
tgaacgccgtcgtgggaaccgccctgatcaaaaagtaccctaagctggaaagcgagttcgtgtacggcgactacaaggtgtacgac
gtgcggaagatgatcgccaagagcgagcaggaaatcggcaaggctaccgccaagtacttcttctacagcaacatcatgaactttttc
aagaccgagattaccctggccaacggcgagatccggaagcggcctctgatcgagacaaacggcgaaaccggggagatcgtgtg
ggataagggccgggattttgccaccgtgcggaaagtgctgagcatgccccaagtgaatatcgtgaaaaagaccgaggtgcagaca
ggcggcttcagcaaagagtctatcctgcccaagaggaacagcgataagctgatcgccagaaagaaggactgggaccctaagaag
tacggcggcttcgacagccccaccgtggcctattctgtgctggtggtggccaaagtggaaaagggcaagtccaagaaactgaagag
tgtgaaagagctgctggggatcaccatcatggaaagaagcagcttcgagaagaatcccatcgactttctggaagccaagggctaca
aagaagtgaaaaaggacctgatcatcaagctgcctaagtactccctgttcgagctggaaaacggccggaagagaatgctggcctct
gccggcgaactgcagaagggaaacgaactggccctgccctccaaatatgtgaacttcctgtacctggccagccactatgagaagct
gaagggctcccccgaggataatgagcagaaacagctgtttgtggaacagcacaagcactacctggacgagatcatcgagcagatc
agcgagttctccaagagagtgatcctggccgacgctaatctggacaaagtgctgtccgcctacaacaagcaccgggataagcccat
cagagagcaggccgagaatatcatccacctgtttaccctgaccaatctgggagcccctgccgccttcaagtactttgacaccaccatc
gaccggaagaggtacaccagcaccaaagaggtgctggacgccaccctgatccaccagagcatcaccggcctgtacgagacacg
gatcgacctgtctcagctgggaggtgactctggcggctcaaaaagaaccgccgacggcagcgaattcgagcccaagaagaagag
gaaagtctaagatctgataatcaacctctggattacaaaatttgtgaaagattgactggtattcttaactatgttgctccttttacgctatgtgg
atacgctgctttaatgcctttgtatcatgctattgcttcccgtatggctttcattttctcctccttgtataaatcctggttagttcttgccacggcgga

actcatcgccgcctgccttgcccgctgctggacaggggctcggctgttgggcactgacaattccgtggtgcgactgtgccttctagttgcc
agccatctgttgtttgcccctcccccgtgccttccttgaccctggaaggtgccactcccactgtcctttcctaataaaatgaggaaattgcat
cgcattgtctgagtaggtgtcattctattctggggggtggggtggggcaggacagcaaggggggaggattgggaagacaatagcagg
catgctggggatgcggtgggctctatggctcgagaaaaaaagcaccgactcggtgccactttttcaagttgataacggactagccttatt
ttaacttgctatttctagctctaAAACTGATAGCGTGATTCTTCCCGCggtgtttcgtcctttccacaagatatataaagcc
aagaaatcgaaatactttcaagttacggtaagcatatgatagtccattttaaaacataattttaaaactgcaaactacccaagaaattatt
actttctacgtcacgtattttgtactaatatctttgtgtttacagtcaaattaattctaattatctctctaacagccttgtatcgtatatgcaaatatg
aaggaatcatgggaaataggccctcttcctgcccgaccttgcggccgcaggaacccctagtgatggagttgg

>pMXsIG.E150K.IRES.GFP, retroviral production for transduction of HEK293T cells
cccgaaaagtgccacctgcataatgaaagaccccacctgtaggtttggcaagctagcttaagtaacgccattttgcaaggcatggaa
aaatacataactgagaatagaaaagttcagatcaaggtcaggaacagatggaacagctgaatatgggccaaacaggatatctgtg
gtaagcagttcctgccccggctcagggccaagaacagatggaacagctgaatatgggccaaacaggatatctgtggtaagcagttc
ctgccccggctcagggccaagaacagatggtccccagatgcggtccagccctcagcagtttctagagaaccatcagatgtttccagg
gtgccccaaggacctgaaatgaccctgtgccttatttgaactaaccaatcagttcgcttctcgcttctgttcgcgcgcttctgctccccgag
ctcaataaaagagcccacaacccctcactcggcgcgccagtcctccgattgactgagtcgcccgggtacccgtgtatccaataaacc
ctcttgcagttgcatccgacttgtggtctcgctgttccttgggagggtctcctctgagtgattgactacccgtcagcgggggtctttcatttgg
gggctcgtccgggatcgggagacccctgcccagggaccaccgacccaccaccgggaggtaagctggccagcaacttatctgtgtct
gtccgattgtctagtgtctatgactgattttatgcgcctgcgtcggtactagttagctaactagctctgtatctggcggacccgtggtggaact
gacgagttcggaacacccggccgcaaccctgggagacgtcccagggacttcgggggccgtttttgtggcccgacctgagtccaaaa
atcccgatcgttttggactctttggtgcacccccctaataggagggatatgtggttctggtaggagacgagaacctaaaacagttcccgc
ctccgtctgaatttttgctttcggtttgggaccgaagccgcgccgcgcgtcttgtctgctgcagcatcgttctgtgttgtctctgtctgactgtgtt
tctgtatttgtctgaaaattagggccagactgttaccactcccttaagtttgaccttaggtcactggaaagatgtcgagcggatcgctcaca
accagtcggtagatgtcaagaagagacgttgggttaccttctgctctgcagaatggccaacctttaacgtcggatggccgcgagacgg
cacctttaaccgagacctcatcacccaggttaagatcaaggtcttttcacctggcccgcatggacacccagaccaggtcccctacatc
gtgacctgggaagccttggcttttgacccccctccctgggtcaagccctttgtacaccctaagcctccgcctcctcttcctccatccgcccc
gtctctcccccttgaacctcctcgttcgaccccgcctcgatcctccctttatccagccctcactccttctctaggcgcccccatatggccata
tgagatcttatatggggcacccccgccccttgtaaacttccctgaccctgacatgacaagagttactaacagcccctctctccaagctca
cttacaggctctctacttagtccagcacgaagtctggagacctctggcggcagcctaccaagaacaactggaccgaccggtggtacc
tcacccttaccgagtcggcgacacagtgtgggtccgccgacaccagactaagaacctagaacctcgctggaaaggaccttacaca
gtcctgctgaccacccccaccgccctcaaagtagacggcatcgcagcttggatacacgccgcccacgtgaaggctgccgaccccg
gggtggaccatcctctagactgccggatctagctagttaattaaggatcccagtgtggtggtacgggaattcagctggacatcacctc
ccacaacgcattgagcgctacgtggtggtctgcaagccgatgagcaacttccgcttcgggaagaatcacgctatcatgggtgtggtctt
cacctggatcatggcgttggcctgtgctgctcccccactcgttggctggtccaggtaatggcactgagtatcgggtctggcaaggtctttgt

gggattccctttgaggacacagagccctcggattggttcctgtccccccagttccagtacgcggccgctacgtaaattccgccccccc
cctcccccccccctaacgttactggccgaagccgcttggaataaggccggtgtgcgtttgtctatatgttattttccaccatattgccgtctttt
ggcaatgtgagggcccggaaacctggccctgtcttcttgacgagcattcctaggggtctttcccctctcgccaaaggaatgcaaggtct
gttgaatgtcgtgaaggaagcagttcctctggaagcttcttgaagacaaacaacgtctgtagcgaccctttgcaggcagcggaacccc
ccacctggcgacaggtgcctctgcggccaaaagccacgtgtataagatacacctgcaaaggcggcacaaccccagtgccacgttg
tgagttggatagttgtggaaagagtcaaatggctctcctcaagcgtattcaacaaggggctgaaggatgcccagaaggtaccccattg
tatgggatctgatctggggcctcggtgcacatgctttacatgtgtttagtcgaggttaaaaaaacgtctaggccccccgaaccacgggg
acgtggttttcctttgaaaaacacgatgataatatggccacaaccatggtgagcaagggcgaggagctgttcaccggggtggtgccc
atcctggtcgagctggacggcgacgtaaacggccacaagttcagcgtgtccggcgagggcgagggcgatgccacctacggcaag
ctgaccctgaagttcatctgcaccaccggcaagctgcccgtgccctggcccaccctcgtgaccaccctgacctacggcgtgcagtgct
tcagccgctaccccgaccacatgaagcagcacgacttcttcaagtccgccatgcccgaaggctacgtccaggagcgcaccatcttct
tcaaggacgacggcaactacaagacccgcgccgaggtgaagttcgagggcgacaccctggtgaaccgcatcgagctgaagggc
atcgacttcaaggaggacggcaacatcctggggcacaagctggagtacaactacaacagccacaacgtctatatcatggccgaca
agcagaagaacggcatcaaggtgaacttcaagatccgccacaacatcgaggacggcagcgtgcagctcgccgaccactaccag
cagaacacccccatcggcgacggccccgtgctgctgcccgacaaccactacctgagcacccagtccgccctgagcaaagacccc
aacgagaagcgcgatcacatggtcctgctggagttcgtgaccgccgccgggatcactctcggcatggacgagctgtacaagtaagt
cgacgataaaataaaagattttatttagtctccagaaaaaggggggaatgaaagaccccacctgtaggtttggcaagctagcttaagt
aacgccattttgcaaggcatggaaaaatacataactgagaatagagaagttcagatcaaggtcaggaacagatggaacagctgaa
tatgggccaaacaggatatctgtggtaagcagttcctgccccggctcagggccaagaacagatggaacagctgaatatgggccaaa
caggatatctgtggtaagcagttcctgccccggctcagggccaagaacagatggtccccagatgcggtccagccctcagcagtttcta
gagaaccatcagatgtttccagggtgccccaaggacctgaaatgaccctgtgccttatttgaactaaccaatcagttcgcttctcgcttct
gttcgcgcgcttctgctccccgagctcaataaaagagcccacaacccctcactcggggcgccagtcctccgattgactgagtcgcccg
ggtacccgtgtatccaataaaccctcttgcagttgcatccgacttgtggtctcgctgttccttgggagggtctcctctgagtgattgactacc
cgtcagcgggggtctttcacatgcagcatgtatcaaaattaatttggttttttttcttaagtatttacattaaatggccatagttgcattaatgaa
tcggccaacgcgcggggagaggcggtttgcgtattgggcgctcttccgcttcctcgctcactgactcgctgcgctcggtcgttcggctgc
ggcgagcggtatcagctcactcaaaggcggtaatacggttatccacagaatcaggggataacgcaggaaagaacatgtgagcaa
aaggccagcaaaaggccaggaaccgtaaaaaggccgcgttgctggcgtttttccataggctccgcccccctgacgagcatcacaa
aaatcgacgctcaagtcagaggtggcgaaacccgacaggactataaagataccaggcgtttccccctggaagctccctcgtgcgct
ctcctgttccgaccctgccgcttaccggatacctgtccgcctttctcccttcgggaagcgtggcgctttctcatagctcacgctgtaggtatc
tcagttcggtgtaggtcgttcgctccaagctgggctgtgtgcacgaaccccccgttcagcccgaccgctgcgccttatccggtaactatc
gtcttgagtccaacccggtaagacacgacttatcgccactggcagcagccactggtaacaggattagcagagcgaggtatgtaggc
ggtgctacagagttcttgaagtggtggcctaactacggctacactagaagaacagtatttggtatctgcgctctgctgaagccagttacct
tcggaaaaagagttggtagctcttgatccggcaaacaaaccaccgctggtagcggtggtttttttgtttgcaagcagcagattacgcgc
agaaaaaaaggatctcaagaagatcctttgatcttttctacggggtctgacgctcagtggaacgaaaactcacgttaagggattttggtc

atgagattatcaaaaaggatcttcacctagatccttttgcggccggccgcaaatcaatctaaagtatatatgagtaaacttggtctgaca
gttaccaatgcttaatcagtgaggcacctatctcagcgatctgtctatttcgttcatccatagttgcctgactccccgtcgtgtagataactac
gatacgggagggcttaccatctggccccagtgctgcaatgataccgcgagacccacgctcaccggctccagatttatcagcaataaa
ccagccagccggaagggccgagcgcagaagtggtcctgcaactttatccgcctccatccagtctattaattgttgccgggaagctaga
gtaagtagttcgccagttaatagtttgcgcaacgttgttgccattgctacaggcatcgtggtgtcacgctcgtcgtttggtatggcttcattca
gctccggttcccaacgatcaaggcgagttacatgatcccccatgttgtgcaaaaaagcggttagctccttcggtcctccgatcgttgtca
gaagtaagttggccgcagtgttatcactcatggttatggcagcactgcataattctcttactgtcatgccatccgtaagatgcttttctgtgac
tggtgagtactcaaccaagtcattctgagaatagtgtatgcggcgaccgagttgctcttgcccggcgtcaatacgggataataccgcgc
cacatagcagaactttaaaagtgctcatcattggaaaacgttcttcggggcgaaaactctcaaggatcttaccgctgttgagatccagtt
cgatgtaacccactcgtgcacccaactgatcttcagcatcttttactttcaccagcgtttctgggtgagcaaaaacaggaaggcaaaat
gccgcaaaaaagggaataagggcgacacggaaatgttgaatactcatactcttcctttttcaatattattgaagcatttatcagggttattg
tctcatgagcggatacatatttgaatgtatttagaaaaataaacaaataggggttccgcgcacatttc

>pCMV.mRho.WT, mammalian expression of WT mRho
ACTCGTGCACCCAACTGATCTTCAGCATCTTTTACTTTCACCAGCGTTTCTGGGTGAGCAAA
AACAGGAAGGCAAAATGCCGCAAAAAAGGGAATAAGGGCGACACGGAAATGTTGAATACT
CATACTCTTCCTTTTTCAATATTATTGAAGCATTTATCAGGGTTATTGTCTCATGAGCGGATA
CATATTTGAATGTATTTAGAAAAATAAACAAATAGGGGTTCCGCGCACATTTCCCCGAAAAG
TGCCACCTGACGTCGACGGATCGGGAGATCGATCTCCCGATCCCCTAGGGTCTACTCTCA
GTACAATCTGCTCTGATGCCGCATAGTTAAGCCAGTATCTGCTCCCTGCTTGTGTGTTGGA
GGTCGCTGAGTAGTGCGCGAGCAAAATTTAAGCTACAACAAGGCAAGGCTTGACCGACAA
TTGCATGAAGAATCTGCTTAGGGTTAGGCGTTTTGCGCTGCTTCGCGATGTACGGGCCAG
ATATACGCGTTGACATTGATTATTGACTAGTTATTAATAGTAATCAATTACGGGGTCATTAGT
TCATAGCCCATATATGGAGTTCCGCGTTACATAACTTACGGTAAATGGCCCGCCTGGCTGA
CCGCCCAACGACCCCCGCCCATTGACGTCAATAATGACGTATGTTCCCATAGTAACGCCAA
TAGGGACTTTCCATTGACGTCAATGGGTGGAGTATTTACGGTAAACTGCCCACTTGGCAGT
ACATCAAGTGTATCATATGCCAAGTACGCCCCCTATTGACGTCAATGACGGTAAATGGCCC
GCCTGGCATTATGCCCAGTACATGACCTTATGGGACTTTCCTACTTGGCAGTACATCTACG
TATTAGTCATCGCTATTACCATGGTGATGCGGTTTTGGCAGTACATCAATGGGCGTGGATA
GCGGTTTGACTCACGGGGATTTCCAAGTCTCCACCCCATTGACGTCAATGGGAGTTTGTTT
TGGCACCAAAATCAACGGGACTTTCCAAAATGTCGTAACAACTCCGCCCCATTGACGCAAA
TGGGCGGTAGGCGTGTACGGTGGGAGGTCTATATAAGCAGAGCTGGTTTAGTGAACCGTC
AGATCCGCTAGAGATCCGCGGCCGCTAATACGACTCACTATAGGGAGAGCCGCCACCATG
AACGGCACAGAGGGCCCCAATTTTTATGTGCCCTTCTCCAACGTCACAGGCGTGGTGCGG

AGCCCCTTCGAGCAGCCGCAGTACTACCTGGCGGAACCATGGCAGTTCTCCATGCTGGCA
GCGTACATGTTCCTGCTCATCGTGCTGGGCTTCCCCATCAACTTCCTCACGCTCTACGTCA
CCGTACAGCACAAGAAGCTGCGCACACCCCTCAACTACATCCTGCTCAACTTGGCCGTGG
CTGACCTCTTCATGGTCTTCGGAGGATTCACCACCACCCTCTACACATCACTCCATGGCTA
CTTCGTCTTTGGGCCCACAGGCTGTAATCTCGAGGGCTTCTTTGCCACACTTGGAGGTGAA
ATCGCCCTGTGGTCCCTGGTGGTCCTGGCCATTGAGCGCTACGTGGTGGTCTGCAAGCCG
ATGAGCAACTTCCGCTTCGGGGAGAATCACGCTATCATGGGTGTGGTCTTCACCTGGATCA
TGGCGTTGGCCTGTGCTGCTCCCCCACTCGTTGGCTGGTCCAGGTACATCCCTGAGGGCA
TGCAATGTTCATGCGGGATTGACTACTACACACTCAAGCCTGAGGTCAACAACGAATCCTT
TGTCATCTACATGTTCGTGGTCCACTTCACCATTCCTATGATCGTCATCTTCTTCTGCTATG
GGCAGCTGGTCTTCACAGTCAAGGAGGCGGCTGCCCAGCAGCAGGAGTCAGCCACCACT
CAGAAGGCAGAGAAGGAAGTCACCCGCATGGTTATCATCATGGTCATCTTCTTCCTGATCT
GCTGGCTTCCCTACGCCAGTGTGGCCTTCTACATCTTCACCCACCAGGGCTCCAACTTCG
GCCCCATCTTCATGACTCTGCCAGCTTTCTTTGCTAAGAGCTCTTCCATCTATAACCCGGTC
ATCTACATCATGTTGAACAAGCAGTTCCGGAACTGTATGCTCACCACGCTGTGCTGCGGCA
AGAATCCACTGGGAGATGACGACGCCTCTGCCACCGCTTCCAAGACGGAGACCAGCCAG
GTGGCTCCAGCCTAACCGGTCATCATCACCATCACCATTGAGTTTAAACCCGCTGATCAGC
CTCGACTGTGCCTTCTAGTTGCCAGCCATCTGTTGTTTGCCCCTCCCCCGTGCCTTCCTTG
ACCCTGGAAGGTGCCACTCCCACTGTCCTTTCCTAATAAAATGAGAAAATTGCATCGCATT
GTCTGAGTAGGTGTCATTCTATTCTGGGGGGTGGGGTGGGGCAGGACAGCAAGGGGGAG
GATTGGGAAGACAATAGCAGGCATGCTGGGGATGCGGTGGGCTCTATGGCTTCTGAGGC
GGAAAGAACCAGCTGGGGCTCGATACCGTCGACCTCTAGCTAGAGCTTGGCGTAATCATG
GTCATAGCTGTTTCCTGTGTGAAATTGTTATCCGCTCACAATTCCACACAACATACGAGCCG
GAAGCATAAAGTGTAAAGCCTAGGGTGCCTAATGAGTGAGCTAACTCACATTAATTGCGTT
GCGCTCACTGCCCGCTTTCCAGTCGGGAAACCTGTCGTGCCAGCTGCATTAATGAATCGG
CCAACGCGCGGGGAGAGGCGGTTTGCGTATTGGGCGCTCTTCCGCTTCCTCGCTCACTGA
CTCGCTGCGCTCGGTCGTTCGGCTGCGGCGAGCGGTATCAGCTCACTCAAAGGCGGTAAT
ACGGTTATCCACAGAATCAGGGGATAACGCAGGAAAGAACATGTGAGCAAAAGGCCAGCA
AAAGGCCAGGAACCGTAAAAAGGCCGCGTTGCTGGCGTTTTTCCATAGGCTCCGCCCCCC
TGACGAGCATCACAAAAATCGACGCTCAAGTCAGAGGTGGCGAAACCCGACAGGACTATA
AAGATACCAGGCGTTTCCCCCTGGAAGCTCCCTCGTGCGCTCTCCTGTTCCGACCCTGCC
GCTTACCGGATACCTGTCCGCCTTTCTCCCTTCGGGAAGCGTGGCGCTTTCTCATAGCTCA
CGCTGTAGGTATCTCAGTTCGGTGTAGGTCGTTCGCTCCAAGCTGGGCTGTGTGCACGAA
CCCCCCGTTCAGCCCGACCGCTGCGCCTTATCCGGTAACTATCGTCTTGAGTCCAACCCG

GTAAGACACGACTTATCGCCACTGGCAGCAGCCACTGGTAACAGGATTAGCAGAGCGAGG
TATGTAGGCGGTGCTACAGAGTTCTTGAAGTGGTGGCCTAACTACGGCTACACTAGAAGAA
CAGTATTTGGTATCTGCGCTCTGCTGAAGCCAGTTACCTTCGGAAAAAGAGTTGGTAGCTC
TTGATCCGGCAAACAAACCACCGCTGGTAGCGGTGGTTTTTTTGTTTGCAAGCAGCAGATT
ACGCGCAGAAAAAAAGGATCTCAAGAAGATCCTTTGATCTTTTCTACGGGGTCTGACACTC
AGTGGAACGAAAACTCACGTTAAGGGATTTTGGTCATGAGATTATCAAAAAGGATCTTCAC
CTAGATCCTTTTAAATTAAAAATGAAGTTTTAAATCAATCTAAAGTATATATGAGTAAACTTG
GTCTGACAGTTACCAATGCTTAATCAGTGAGGCACCTATCTCAGCGATCTGTCTATTTCGTT
CATCCATAGTTGCCTGACTCCCCGTCGTGTAGATAACTACGATACGGGAGGGCTTACCATC
TGGCCCCAGTGCTGCAATGATACCGCGAGACCCACGCTCACCGGCTCCAGATTTATCAGC
AATAAACCAGCCAGCCGGAAGGGCCGAGCGCAGAAGTGGTCCTGCAACTTTATCCGCCTC
CATCCAGTCTATTAATTGTTGCCGGGAAGCTAGAGTAAGTAGTTCGCCAGTTAATAGTTTGC
GCAACGTTGTTGCCATTGCTACAGGCATCGTGGTGTCACGCTCGTCGTTTGGTATGGCTTC
ATTCAGCTCCGGTTCCCAACGATCAAGGCGAGTTACATGATCCCCCATGTTGTGCAAAAAA
GCGGTTAGCTCCTTCGGTCCTCCGATCGTTGTCAGAAGTAAGTTGGCCGCAGTGTTATCAC
TCATGGTTATGGCAGCACTGCATAATTCTCTTACTGTCATGCCATCCGTAAGATGCTTTTCT
GTGACTGGTGAGTACTCAACCAAGTCATTCTGAGAATAGTGTATGCGGCGACCGAGTTGCT
CTTGCCCGGCGTCAATACGGGATAATACCGCGCCACATAGCAGAACTTTAAAAGTGCTCAT
CATTGGAAAACGTTCTTCGGGGCGAAAACTCTCAAGGATCTTACCGCTGTTGAGATCCAGT
TCGATGTAACCC

>pCMV.mRho.E150K, mammalian expression of E150K mRho
ACTCGTGCACCCAACTGATCTTCAGCATCTTTTACTTTCACCAGCGTTTCTGGGTGAGCAAA
AACAGGAAGGCAAAATGCCGCAAAAAAGGGAATAAGGGCGACACGGAAATGTTGAATACT
CATACTCTTCCTTTTTCAATATTATTGAAGCATTTATCAGGGTTATTGTCTCATGAGCGGATA
CATATTTGAATGTATTTAGAAAAATAAACAAATAGGGGTTCCGCGCACATTTCCCCGAAAAG
TGCCACCTGACGTCGACGGATCGGGAGATCGATCTCCCGATCCCCTAGGGTCTACTCTCA
GTACAATCTGCTCTGATGCCGCATAGTTAAGCCAGTATCTGCTCCCTGCTTGTGTGTTGGA
GGTCGCTGAGTAGTGCGCGAGCAAAATTTAAGCTACAACAAGGCAAGGCTTGACCGACAA
TTGCATGAAGAATCTGCTTAGGGTTAGGCGTTTTGCGCTGCTTCGCGATGTACGGGCCAG
ATATACGCGTTGACATTGATTATTGACTAGTTATTAATAGTAATCAATTACGGGGTCATTAGT
TCATAGCCCATATATGGAGTTCCGCGTTACATAACTTACGGTAAATGGCCCGCCTGGCTGA
CCGCCCAACGACCCCCGCCCATTGACGTCAATAATGACGTATGTTCCCATAGTAACGCCAA
TAGGGACTTTCCATTGACGTCAATGGGTGGAGTATTTACGGTAAACTGCCCACTTGGCAGT

ACATCAAGTGTATCATATGCCAAGTACGCCCCCTATTGACGTCAATGACGGTAAATGGCCC
GCCTGGCATTATGCCCAGTACATGACCTTATGGGACTTTCCTACTTGGCAGTACATCTACG
TATTAGTCATCGCTATTACCATGGTGATGCGGTTTTGGCAGTACATCAATGGGCGTGGATA
GCGGTTTGACTCACGGGGATTTCCAAGTCTCCACCCCATTGACGTCAATGGGAGTTTGTTT
TGGCACCAAAATCAACGGGACTTTCCAAAATGTCGTAACAACTCCGCCCCATTGACGCAAA
TGGGCGGTAGGCGTGTACGGTGGGAGGTCTATATAAGCAGAGCTGGTTTAGTGAACCGTC
AGATCCGCTAGAGATCCGCGGCCGCTAATACGACTCACTATAGGGAGAGCCGCCACCATG
AACGGCACAGAGGGCCCCAATTTTTATGTGCCCTTCTCCAACGTCACAGGCGTGGTGCGG
AGCCCCTTCGAGCAGCCGCAGTACTACCTGGCGGAACCATGGCAGTTCTCCATGCTGGCA
GCGTACATGTTCCTGCTCATCGTGCTGGGCTTCCCCATCAACTTCCTCACGCTCTACGTCA
CCGTACAGCACAAGAAGCTGCGCACACCCCTCAACTACATCCTGCTCAACTTGGCCGTGG
CTGACCTCTTCATGGTCTTCGGAGGATTCACCACCACCCTCTACACATCACTCCATGGCTA
CTTCGTCTTTGGGCCCACAGGCTGTAATCTCGAGGGCTTCTTTGCCACACTTGGAGGTGAA
ATCGCCCTGTGGTCCCTGGTGGTCCTGGCCATTGAGCGCTACGTGGTGGTCTGCAAGCCG
ATGAGCAACTTCCGCTTCGGGAAGAATCACGCTATCATGGGTGTGGTCTTCACCTGGATCA
TGGCGTTGGCCTGTGCTGCTCCCCCACTCGTTGGCTGGTCCAGGTACATCCCTGAGGGCA
TGCAATGTTCATGCGGGATTGACTACTACACACTCAAGCCTGAGGTCAACAACGAATCCTT
TGTCATCTACATGTTCGTGGTCCACTTCACCATTCCTATGATCGTCATCTTCTTCTGCTATG
GGCAGCTGGTCTTCACAGTCAAGGAGGCGGCTGCCCAGCAGCAGGAGTCAGCCACCACT
CAGAAGGCAGAGAAGGAAGTCACCCGCATGGTTATCATCATGGTCATCTTCTTCCTGATCT
GCTGGCTTCCCTACGCCAGTGTGGCCTTCTACATCTTCACCCACCAGGGCTCCAACTTCG
GCCCCATCTTCATGACTCTGCCAGCTTTCTTTGCTAAGAGCTCTTCCATCTATAACCCGGTC
ATCTACATCATGTTGAACAAGCAGTTCCGGAACTGTATGCTCACCACGCTGTGCTGCGGCA
AGAATCCACTGGGAGATGACGACGCCTCTGCCACCGCTTCCAAGACGGAGACCAGCCAG
GTGGCTCCAGCCTAACCGGTCATCATCACCATCACCATTGAGTTTAAACCCGCTGATCAGC
CTCGACTGTGCCTTCTAGTTGCCAGCCATCTGTTGTTTGCCCCTCCCCCGTGCCTTCCTTG
ACCCTGGAAGGTGCCACTCCCACTGTCCTTTCCTAATAAAATGAGAAATTGCATCGCATT
GTCTGAGTAGGTGTCATTCTATTCTGGGGGGTGGGGTGGGGCAGGACAGCAAGGGGGAG
GATTGGGAAGACAATAGCAGGCATGCTGGGGATGCGGTGGGCTCTATGGCTTCTGAGGC
GGAAAGAACCAGCTGGGGCTCGATACCGTCGACCTCTAGCTAGAGCTTGGCGTAATCATG
GTCATAGCTGTTTCCTGTGTGAAATTGTTATCCGCTCACAATTCCACACAACATACGAGCCG
GAAGCATAAAGTGTAAAGCCTAGGGTGCCTAATGAGTGAGCTAACTCACATTAATTGCGTT
GCGCTCACTGCCCGCTTTCCAGTCGGGAAACCTGTCGTGCCAGCTGCATTAATGAATCGG
CCAACGCGCGGGGAGAGGCGGTTTGCGTATTGGGCGCTCTTCCGCTTCCTCGCTCACTGA

CTCGCTGCGCTCGGTCGTTCGGCTGCGGCGAGCGGTATCAGCTCACTCAAAGGCGGTAAT
ACGGTTATCCACAGAATCAGGGGATAACGCAGGAAAGAACATGTGAGCAAAAGGCCAGCA
AAAGGCCAGGAACCGTAAAAAGGCCGCGTTGCTGGCGTTTTTCCATAGGCTCCGCCCCCC
TGACGAGCATCACAAAAATCGACGCTCAAGTCAGAGGTGGCGAAACCCGACAGGACTATA
AAGATACCAGGCGTTTCCCCCTGGAAGCTCCCTCGTGCGCTCTCCTGTTCCGACCCTGCC
GCTTACCGGATACCTGTCCGCCTTTCTCCCTTCGGGAAGCGTGGCGCTTTCTCATAGCTCA
CGCTGTAGGTATCTCAGTTCGGTGTAGGTCGTTCGCTCCAAGCTGGGCTGTGTGCACGAA
CCCCCCGTTCAGCCCGACCGCTGCGCCTTATCCGGTAACTATCGTCTTGAGTCCAACCCG
GTAAGACACGACTTATCGCCACTGGCAGCAGCCACTGGTAACAGGATTAGCAGAGCGAGG
TATGTAGGCGGTGCTACAGAGTTCTTGAAGTGGTGGCCTAACTACGGCTACACTAGAAGAA
CAGTATTTGGTATCTGCGCTCTGCTGAAGCCAGTTACCTTCGGAAAAAGAGTTGGTAGCTC
TTGATCCGGCAAACAAACCACCGCTGGTAGCGGTGGTTTTTTTGTTTGCAAGCAGCAGATT
ACGCGCAGAAAAAAAGGATCTCAAGAAGATCCTTTGATCTTTTCTACGGGGTCTGACACTC
AGTGGAACGAAAACTCACGTTAAGGGATTTTGGTCATGAGATTATCAAAAAGGATCTTCAC
CTAGATCCTTTTAAATTAAAAATGAAGTTTTAAATCAATCTAAAGTATATATGAGTAAACTTG
GTCTGACAGTTACCAATGCTTAATCAGTGAGGCACCTATCTCAGCGATCTGTCTATTTCGTT
CATCCATAGTTGCCTGACTCCCCGTCGTGTAGATAACTACGATACGGGAGGGCTTACCATC
TGGCCCCAGTGCTGCAATGATACCGCGAGACCCACGCTCACCGGCTCCAGATTTATCAGC
AATAAACCAGCCAGCCGGAAGGGCCGAGCGCAGAAGTGGTCCTGCAACTTTATCCGCCTC
CATCCAGTCTATTAATTGTTGCCGGGAAGCTAGAGTAAGTAGTTCGCCAGTTAATAGTTTGC
GCAACGTTGTTGCCATTGCTACAGGCATCGTGGTGTCACGCTCGTCGTTTGGTATGGCTTC
ATTCAGCTCCGGTTCCCAACGATCAAGGCGAGTTACATGATCCCCCATGTTGTGCAAAAAA
GCGGTTAGCTCCTTCGGTCCTCCGATCGTTGTCAGAAGTAAGTTGGCCGCAGTGTTATCAC
TCATGGTTATGGCAGCACTGCATAATTCTCTTACTGTCATGCCATCCGTAAGATGCTTTTCT
GTGACTGGTGAGTACTCAACCAAGTCATTCTGAGAATAGTGTATGCGGCGACCGAGTTGCT
CTTGCCCGGCGTCAATACGGGATAATACCGCGCCACATAGCAGAACTTTAAAAGTGCTCAT
CATTGGAAAACGTTCTTCGGGGCGAAAACTCTCAAGGATCTTACCGCTGTTGAGATCCAGT
TCGATGTAACCC

>pCMV.ABEmax, mammalian expression of ABEmax, Addgene #112095
atatgccaagtacgccccctattgacgtcaatgacggtaaatggcccgcctggcattatgcccagtacatgaccttatgggactttccta
cttggcagtacatctacgtattagtcatcgctattaccatggtgatgcggttttggcagtacatcaatgggcgtggatagcggtttgactca
cggggatttccaagtctccaccccattgacgtcaatgggagtttgttttggcaccaaaatcaacgggactttccaaaatgtcgtaacaac
tccgccccattgacgcaaatgggcggtaggcgtgtacggtgggaggtctatataagcagagctggtttagtgaaccgtcagatccgct

agagatccgcggccgctaatacgactcactatagggagagccgccaccatgaaacggacagccgacggaagcgagttcgagtc
accaaagaagaagcggaaagtctctgaagtcgagtttagccacgagtattggatgaggcacgcactgaccctggcaaagcgagc
atgggatgaaagagaagtccccgtgggcgccgtgctggtgcacaacaatagagtgatcggagagggatggaacaggccaatcgg
ccgccacgaccctaccgcacacgcagagatcatggcactgaggcagggaggcctggtcatgcagaattaccgcctgatcgatgcc
accctgtatgtgacactggagccatgcgtgatgtgcgcaggagcaatgatccacagcaggatcggaagagtggtgttcggagcacg
ggacgccaagaccggcgcagcaggctccctgatggatgtgctgcaccaccccggcatgaaccaccgggtggagatcacagagg
gaatcctggcagacgagtgcgccgccctgctgagcgatttctttagaatgcggagacaggagatcaaggcccagaagaaggcaca
gagctccaccgactctggaggatctagcggaggatcctctggaagcgagacaccaggcacaagcgagtccgccacaccagaga
gctccggcggctcctccggaggatcctctgaggtggagttttcccacgagtactggatgagacatgccctgaccctggccaagaggg
cacgcgatgagagggaggtgcctgtgggagccgtgctggtgctgaacaatagagtgatcggcgagggctggaacagagccatcg
gcctgcacgacccaacagcccatgccgaaattatggccctgagacagggcggcctggtcatgcagaactacagactgattgacgc
caccctgtacgtgacattcgagccttgcgtgatgtgcgccggcgccatgatccactctaggatcggccgcgtggtgtttggcgtgagga
acgcaaaaaccggcgccgcaggctccctgatggacgtgctgcactaccccggcatgaatcaccgcgtcgaaattaccgagggaat
cctggcagatgaatgtgccgccctgctgtgctatttctttcggatgcctagacaggtgttcaatgctcagaagaaggcccagagctccac
cgactccggaggatctagcggaggctcctctggctctgagacacctggcacaagcgagagcgcaacacctgaaagcagcggggg
cagcagcgggggggtcagacaagaagtacagcatcggcctggccatcggcaccaactctgtgggctgggccgtgatcaccgacga
gtacaaggtgcccagcaagaaattcaaggtgctgggcaacaccgaccggcacagcatcaagaagaacctgatcggagccctgct
gttcgacagcggcgaaacagccgaggccacccggctgaagagaaccgccagaagaagatacaccagacggaagaaccggat
ctgctatctgcaagagatcttcagcaacgagatggccaaggtggacgacagcttcttccacagactggaagagtccttcctggtggaa
gaggataagaagcacgagcggcaccccatcttcggcaacatcgtggacgaggtggcctaccacgagaagtaccccaccatctac
cacctgagaaagaaactggtggacagcaccgacaaggccgacctgcggctgatctatctggccctggcccacatgatcaagttccg
gggccacttcctgatcgagggcgacctgaaccccgacaacagcgacgtggacaagctgttcatccagctggtgcagacctacaac
cagctgttcgaggaaaaccccatcaacgccagcggcgtggacgccaaggccatcctgtctgccagactgagcaagagcagacgg
ctggaaaatctgatcgcccagctgcccggcgagaagaagaatggcctgttcggaaacctgattgccctgagcctgggcctgacccc
caacttcaagagcaacttcgacctggccgaggatgccaaactgcagctgagcaaggacacctacgacgacgacctggacaacct
gctggcccagatcggcgaccagtacgccgacctgtttctggccgccaagaacctgtccgacgccatcctgctgagcgacatcctgag
agtgaacaccgagatcaccaaggcccccctgagcgcctctatgatcaagagatacgacgagcaccaccaggacctgaccctgctg
aaagctctcgtgcggcagcagctgcctgagaagtacaaagagattttcttcgaccagagcaagaacggctacgccggctacattga
cggcggagccagccaggaagagttctacaagttcatcaagcccatcctggaaaagatggacggcaccgaggaactgctcgtgaa
gctgaacagagaggacctgctgcggaagcagcggaccttcgacaacggcagcatcccccaccagatccacctgggagagctgc
acgccattctgcggcggcaggaagatttttacccattcctgaaggacaaccgggaaaagatcgagaagatcctgaccttccgcatcc
cctactacgtgggccctctggccaggggaaacagcagattcgcctggatgaccagaaagagcgaggaaaccatcacccccctgga
acttcgaggaagtggtggacaagggcgcttccgcccagagcttcatcgagcggatgaccaacttcgataagaacctgcccaacga
gaaggtgctgcccaagcacagcctgctgtacgagtacttcaccgtgtataacgagctgaccaaagtgaaatacgtgaccgagggaa

tgagaaagcccgccttcctgagcggcgagcagaaaaaggccatcgtggacctgctgttcaagaccaaccggaaagtgaccgtga
agcagctgaaagaggactacttcaagaaaatcgagtgcttcgactccgtggaaatctccggcgtggaagatcggttcaacgcctccc
tgggcacataccacgatctgctgaaaattatcaaggacaaggacttcctggacaatgaggaaaacgaggacattctggaagatatc
gtgctgaccctgacactgtttgaggacagagagatgatcgaggaacggctgaaaacctatgcccacctgttcgacgacaaagtgatg
aagcagctgaagcggcggagatacaccggctggggcaggctgagccggaagctgatcaacggcatccgggacaagcagtccg
gcaagacaatcctggatttcctgaagtccgacggcttcgccaacagaaacttcatgcagctgatccacgacgacagcctgacctttaa
agaggacatccagaaagcccaggtgtccggccagggcgatagcctgcacgagcacattgccaatctggccggcagccccgccat
taagaagggcatcctgcagacagtgaaggtggtggacgagctcgtgaaagtgatgggccggcacaagcccgagaacatcgtgat
cgaaatggccagagagaaccagaccacccagaagggacagaagaacagccgcgagagaatgaagcggatcgaagagggc
atcaaagagctgggcagccagatcctgaaagaacaccccgtggaaaacacccagctgcagaacgagaagctgtacctgtactac
ctgcagaatgggcgggatatgtacgtggaccaggaactggacatcaaccggctgtccgactacgatgtggaccatatcgtgcctcag
agctttctgaaggacgactccatcgacaacaaggtgctgaccagaagcgacaagaaccggggcaagagcgacaacgtgccctcc
gaagaggtcgtgaagaagatgaagaactactggcggcagctgctgaacgccaagctgattacccagagaaagttcgacaatctga
ccaaggccgagagaggcggcctgagcgaactggataaggccggcttcatcaagagacagctggtggaaacccggcagatcaca
aagcacgtggcacagatcctggactcccggatgaacactaagtacgacgagaatgacaagctgatccgggaagtgaaagtgatc
accctgaagtccaagctggtgtccgatttccggaaggatttccagttttacaaagtgcgcgagatcaacaactaccaccacgcccacg
acgcctacctgaacgccgtcgtgggaaccgccctgatcaaaaagtaccctaagctggaaagcgagttcgtgtacggcgactacaag
gtgtacgacgtgcggaagatgatcgccaagagcgagcaggaaatcggcaaggctaccgccaagtacttcttctacagcaacatcat
gaactttttcaagaccgagattaccctggccaacggcgagatccggaagcggcctctgatcgagacaaacggcgaaaccgggga
gatcgtgtgggataagggccgggattttgccaccgtgcggaaagtgctgagcatgccccaagtgaatatcgtgaaaaagaccgagg
tgcagacaggcggcttcagcaaagagtctatcctgcccaagaggaacagcgataagctgatcgccagaaagaaggactgggacc
ctaagaagtacggcggcttcgacagccccaccgtggcctattctgtgctggtggtggccaaagtggaaaagggcaagtccaagaaa
ctgaagagtgtgaaagagctgctggggatcaccatcatggaaagaagcagcttcgagaagaatcccatcgactttctggaagccaa
gggctacaaagaagtgaaaaaggacctgatcatcaagctgcctaagtactccctgttcgagctggaaaacggccggaagagaatg
ctggcctctgccggcgaactgcagaagggaaacgaactggccctgccctccaaatatgtgaacttcctgtacctggccagccactat
gagaagctgaagggctcccccgaggataatgagcagaaacagctgtttgtggaacagcacaagcactacctggacgagatcatcg
agcagatcagcgagttctccaagagagtgatcctggccgacgctaatctggacaaagtgctgtccgcctacaacaagcaccgggat
aagcccatcagagagcaggccgagaatatcatccacctgtttaccctgaccaatctgggagcccctgccgccttcaagtactttgaca
ccaccatcgaccggaagaggtacaccagcaccaaagaggtgctggacgccaccctgatccaccagagcatcaccggcctgtacg
agacacggatcgacctgtctcagctgggaggtgactctggcggctcaaaaagaaccgccgacggcagcgaattcgagcccaaga
agaagaggaaagtctaaccggtcatcatcaccatcaccattgagtttaaacccgctgatcagcctcgactgtgccttctagttgccagc
catctgttgtttgcccctcccccgtgccttccttgaccctggaaggtgccactcccactgtcctttcctaataaaatgaggaaattgcatcgc
attgtctgagtaggtgtcattctattctggggggtggggtggggcaggacagcaagggggaggattgggaagacaatagcaggcatg
ctggggatgcggtgggctctatggcttctgaggcggaaagaaccagctggggctcgataccgtcgacctctagctagagcttggcgta

atcatggtcatagctgtttcctgtgtgaaattgttatccgctcacaattccacacaacatacgagccggaagcataaagtgtaaagccta
gggtgcctaatgagtgagctaactcacattaattgcgttgcgctcactgcccgctttccagtcgggaaacctgtcgtgccagctgcattaa
tgaatcggccaacgcgcggggagaggcggtttgcgtattgggcgctcttccgcttcctcgctcactgactcgctgcgctcggtcgttcgg
ctgcggcgagcggtatcagctcactcaaaggcggtaatacggttatccacagaatcaggggataacgcaggaaagaacatgtgag
caaaaggccagcaaaaggccaggaaccgtaaaaaggccgcgttgctggcgtttttccataggctccgcccccctgacgagcatca
caaaaatcgacgctcaagtcagaggtggcgaaacccgacaggactataaagataccaggcgtttccccctggaagctccctcgtgc
gctctcctgttccgaccctgccgcttaccggatacctgtccgcctttctcccttcgggaagcgtggcgctttctcatagctcacgctgtaggt
atctcagttcggtgtaggtcgttcgctccaagctgggctgtgtgcacgaaccccccgttcagcccgaccgctgcgccttatccggtaact
atcgtcttgagtccaacccggtaagacacgacttatcgccactggcagcagccactggtaacaggattagcagagcgaggtatgtag
gcggtgctacagagttcttgaagtggtggcctaactacggctacactagaagaacagtatttggtatctgcgctctgctgaagccagtta
ccttcggaaaaagagttggtagctcttgatccggcaaacaaaccaccgctggtagcggtggtttttttgtttgcaagcagcagattacgc
gcagaaaaaaaggatctcaagaagatcctttgatcttttctacggggtctgacactcagtggaacgaaaactcacgttaagggattttg
gtcatgagattatcaaaaaggatcttcacctagatccttttaaattaaaaatgaagttttaaatcaatctaaagtatatatgagtaaacttgg
tctgacagttaccaatgcttaatcagtgaggcacctatctcagcgatctgtctatttcgttcatccatagttgcctgactccccgtcgtgtaga
taactacgatacgggagggcttaccatctggccccagtgctgcaatgataccgcgagacccacgctcaccggctccagatttatcag
caataaaccagccagccggaagggccgagcgcagaagtggtcctgcaactttatccgcctccatccagtctattaattgttgccggga
agctagagtaagtagttcgccagttaatagtttgcgcaacgttgttgccattgctacaggcatcgtggtgtcacgctcgtcgtttggtatgg
cttcattcagctccggttcccaacgatcaaggcgagttacatgatcccccatgttgtgcaaaaaagcggttagctccttcggtcctccgat
cgttgtcagaagtaagttggccgcagtgttatcactcatggttatggcagcactgcataattctcttactgtcatgccatccgtaagatgcttt
tctgtgactggtgagtactcaaccaagtcattctgagaatagtgtatgcggcgaccgagttgctcttgcccggcgtcaatacgggataat
accgcgccacatagcagaactttaaaagtgctcatcattggaaaacgttcttcggggcgaaaactctcaaggatcttaccgctgttgag
atccagttcgatgtaacccactcgtgcacccaactgatcttcagcatcttttactttcaccagcgtttctgggtgagcaaaaacaggaagg
caaaatgccgcaaaaaagggaataagggcgacacggaaatgttgaatactcatactcttcctttttcaatattattgaagcatttatcag
ggttattgtctcatgagcggatacatatttgaatgtatttagaaaaataaacaaataggggttccgcgcacatttccccgaaaagtgcca
cctgacgtcgacggatcgggagatcgatctcccgatcccctagggtcgactctcagtacaatctgctctgatgccgcatagttaagcca
gtatctgctccctgcttgtgtgttggaggtcgctgagtagtgcgcgagcaaaatttaagctacaacaaggcaaggcttgaccgacaatt
gcatgaagaatctgcttagggttaggcgttttgcgctgcttcgcgatgtacgggccagatatacgcgttgacattgattattgactagttatt
aatagtaatcaattacggggtcattagttcatagcccatatatggagttccgcgttacataacttacggtaaatggcccgcctggctgacc
gcccaacgacccccgcccattgacgtcaataatgacgtatgttcccatagtaacgccaatagggactttccattgacgtcaatgggtgg
agtatttacggtaaactgcccacttggcagtacatcaagtgtatc

>pCMV.PE2.SpRY, mammalian expression of PE2-SpRY, Addgene #159979
gacattgattattgactagttattaatagtaatcaattacggggtcattagttcatagcccatatatggagttccgcgttacataacttacggt
aaatggcccgcctggctgaccgcccaacgacccccgcccattgacgtcaataatgacgtatgttcccatagtaacgccaatagggac

tttccattgacgtcaatgggtggagtatttacggtaaactgcccacttggcagtacatcaagtgtatcatatgccaagtacgccccctattg
acgtcaatgacggtaaatggcccgcctggcattatgcccagtacatgaccttatgggactttcctacttggcagtacatctacgtattagt
catcgctattaccatggtgatgcggttttggcagtacatcaatgggcgtggatagcggtttgactcacggggatttccaagtctccacccc
attgacgtcaatgggagtttgttttggcaccaaaatcaacgggactttccaaaatgtcgtaacaactccgccccattgacgcaaatggg
cggtaggcgtgtacggtgggaggtctatataagcagagctggtttagtgaaccgtcagatccgctagagatccgcggccgctaatac
gactcactatagggagagccgccaccatgaaacggacagccgacggaagcgagttcgagtcaccaaagaagaagcggaaagt
cgacaagaagtacagcatcggcctggacatcggcaccaactctgtgggctgggccgtgatcaccgacgagtacaaggtgcccagc
aagaaattcaaggtgctgggcaacaccgaccggcacagcatcaagaagaacctgatcggagccctgctgttcgacagcggcgaa
acagccgagagaacccggctgaagagaaccgccagaagaagatacaccagacggaagaaccggatctgctatctgcaagaga
tcttcagcaacgagatggccaaggtggacgacagcttcttccacagactggaagagtccttcctggtggaagaggataagaagcac
gagcggcaccccatcttcggcaacatcgtggacgaggtggcctaccacgagaagtaccccaccatctaccacctgagaaagaaa
ctggtggacagcaccgacaaggccgacctgcggctgatctatctggccctggcccacatgatcaagttccggggccacttcctgatcg
agggcgacctgaaccccgacaacagcgacgtggacaagctgttcatccagctggtgcagacctacaaccagctgttcgaggaaaa
ccccatcaacgccagcggcgtggacgccaaggccatcctgtctgccagactgagcaagagcagacggctggaaaatctgatcgc
ccagctgcccggcgagaagaagaatggcctgttcggaaacctgattgccctgagcctgggcctgacccccaacttcaagagcaact
tcgacctggccgaggatgccaaactgcagctgagcaaggacacctacgacgacgacctggacaacctgctggcccagatcggcg
accagtacgccgacctgtttctggccgccaagaacctgtccgacgccatcctgctgagcgacatcctgagagtgaacaccgagatca
ccaaggcccccctgagcgcctctatgatcaagagatacgacgagcaccaccaggacctgaccctgctgaaagctctcgtgcggca
gcagctgcctgagaagtacaaagagattttcttcgaccagagcaagaacggctacgccggctacattgacggcggagccagccag
gaagagttctacaagttcatcaagcccatcctggaaaagatggacggcaccgaggaactgctcgtgaagctgaacagagaggacc
tgctgcggaagcagcggaccttcgacaacggcagcatcccccaccagatccacctgggagagctgcacgccattctgcggcggca
ggaagatttttacccattcctgaaggacaaccgggaaaagatcgagaagatcctgaccttccgcatcccctactacgtgggccctctg
gccaggggaaacagcagattcgcctggatgaccagaaagagcgaggaaaccatcacccccctggaacttcgaggaagtggtgga
caagggcgcttccgcccagagcttcatcgagcggatgaccaacttcgataagaacctgcccaacgagaaggtgctgcccaagcac
agcctgctgtacgagtacttcaccgtgtataacgagctgaccaaagtgaaatacgtgaccgagggaatgagaaagcccgccttcctg
agcggcgagcagaaaaaggccatcgtggacctgctgttcaagaccaaccggaaagtgaccgtgaagcagctgaaagaggacta
cttcaagaaaatcgagtgcttcgactccgtggaaatctccggcgtggaagatcggttcaacgcctccctgggcacataccacgatctg
ctgaaaattatcaaggacaaggacttcctggacaatgaggaaaacgaggacattctggaagatatcgtgctgaccctgacactgtttg
aggacagagagatgatcgaggaacggctgaaaacctatgcccacctgttcgacgacaaagtgatgaagcagctgaagcggcgg
agatacaccggctggggcaggctgagccggaagctgatcaacggcatccgggacaagcagtccggcaagacaatcctggatttc
ctgaagtccgacggcttcgccaacagaaacttcatgcagctgatccacgacgacagcctgacctttaaagaggacatccagaaagc
ccaggtgtccggccagggcgatagcctgcacgagcacattgccaatctggccggcagccccgccattaagaagggcatcctgcag
acagtgaaggtggtggacgagctcgtgaaagtgatgggccggcacaagcccgagaacatcgtgatcgaaatggccagagagaa
ccagaccacccagaagggacagaagaacagccgcgagagaatgaagcggatcgaagagggcatcaaagagctgggcagcc

agatcctgaaagaacaccccgtggaaaacacccagctgcagaacgagaagctgtacctgtactacctgcagaatgggcgggatat
gtacgtggaccaggaactggacatcaaccggctgtccgactacgatgtggacgctatcgtgcctcagagctttctgaaggacgactcc
atcgacaacaaggtgctgaccagaagcgacaagaaccggggcaagagcgacaacgtgccctccgaagaggtcgtgaagaaga
tgaagaactactggcggcagctgctgaacgccaagctgattacccagagaaagttcgacaatctgaccaaggccgagagaggcg
gcctgagcgaactggataaggccggcttcatcaagagacagctggtggaaacccggcagatcacaaagcacgtggcacagatcc
tggactcccggatgaacactaagtacgacgagaatgacaagctgatccgggaagtgaaagtgatcaccctgaagtccaagctggt
gtccgatttccggaaggatttccagttttacaaagtgcgcgagatcaacaactaccaccacgcccacgacgcctacctgaacgccgtc
gtgggaaccgccctgatcaaaaagtaccctaagctggaaagcgagttcgtgtacggcgactacaaggtgtacgacgtgcggaaga
tgatcgccaagagcgagcaggaaatcggcaaggctaccgccaagtacttcttctacagcaacatcatgaactttttcaagaccgaga
ttaccctggccaacggcgagatccggaagcggcctctgatcgagacaaacggcgaaaccggggagatcgtgtgggataagggcc
gggattttgccaccgtgcggaaagtgctgagcatgccccaagtgaatatcgtgaaaaagaccgaggtgcagacaggcggcttcagc
aaagagtctatcagacccaagaggaacagcgataagctgatcgccagaaagaaggactgggaccctaagaagtacggcggctt
cctgtggcccaccgtggcctattctgtgctggtggtggccaaagtggaaaagggcaagtccaagaaactgaagagtgtgaaagagc
tgctggggatcaccatcatggaaagaagcagcttcgagaagaatcccatcgactttctggaagccaagggctacaaagaagtgaa
aaaggacctgatcatcaagctgcctaagtactccctgttcgagctggaaaacggccggaagagaatgctggcctctgccaagcagc
tgcagaagggaaacgaactggccctgccctccaaatatgtgaacttcctgtacctggccagccactatgagaagctgaagggctccc
ccgaggataatgagcagaaacagctgtttgtggaacagcacaagcactacctggacgagatcatcgagcagatcagcgagttctcc
aagagagtgatcctggccgacgctaatctggacaaagtgctgtccgcctacaacaagcaccgggataagcccatcagagagcag
gccgagaatatcatccacctgtttaccctgaccagactgggagcccctagagccttcaagtactttgacaccaccatcgaccccaagc
agtacagaagcaccaaagaggtgctggacgccaccctgatccaccagagcatcaccggcctgtacgagacacggatcgacctgt
ctcagctgggaggtgactctggaggatctagcggaggatcctctggcagcgagacaccaggaacaagcgagtcagcaacaccag
agagcagtggcggcagcagcggcggcagcagcaccctaaatatagaagatgagtatcggctacatgagacctcaaaagagcca
gatgtttctctagggtccacatggctgtctgattttcctcaggcctgggcggaaaccgggggcatgggactggcagttcgccaagctcct
ctgatcatacctctgaaagcaacctctacccccgtgtccataaaacaataccccatgtcacaagaagccagactggggatcaagccc
cacatacagagactgttggaccagggaatactggtaccctgccagtcccccctggaacacgcccctgctacccgttaagaaaccagg
gactaatgattataggcctgtccaggatctgagagaagtcaacaagcgggtggaagacatccaccccaccgtgcccaacccttaca
acctcttgagcgggctcccaccgtcccaccagtggtacactgtgcttgatttaaaggatgcctttttctgcctgagactccaccccaccag
tcagcctctcttcgcctttgagtggagagatccagagatgggaatctcaggacaattgacctggaccagactcccacagggtttcaaa
aacagtcccaccctgtttaatgaggcactgcacagagacctagcagacttccggatccagcacccagacttgatcctgctacagtac
gtggatgacttactgctggccgccacttctgagctagactgccaacaaggtactcgggccctgttacaaaccctagggaacctcgggt
atcgggcctcggccaagaaagcccaaatttgccagaaacaggtcaagtatctggggtatcttctaaaagagggtcagagatggctg
actgaggccagaaaagagactgtgatggggcagcctactccgaagacccctcgacaactaagggagttcctagggaaggcaggc
ttctgtcgcctcttcatccctgggtttgcagaaatggcagccccctgtaccctctcaccaaaccggggactctgtttaattggggcccag
accaacaaaaggcctatcaagaaatcaagcaagctcttctaactgccccagccctggggttgccagatttgactaagccctttgaact

ctttgtcgacgagaagcagggctacgccaaaggtgtcctaacgcaaaaactgggaccttggcgtcggccggtggcctacctgtccaa
aaagctagacccagtagcagctgggtggccccttgcctacggatggtagcagccattgccgtactgacaaaggatgcaggcaag
ctaaccatgggacagccactagtcattctggccccccatgcagtagaggcactagtcaaacaacccccgaccgctggctttccaac
gcccggatgactcactatcaggccttgcttttggacacggaccgggtccagttcggaccggtggtagccctgaacccggctacgctgc
tcccactgcctgaggaagggctgcaacacaactgccttgatatcctggccgaagcccacggaacccgacccgacctaacggacca
gccgctcccagacgccgaccacacctggtacacggatggaagcagtctcttacaagagggacagcgtaaggcgggagctgcggt
gaccaccgagaccgaggtaatctgggctaaagccctgccagccgggacatccgctcagcgggctgaactgatagcactcaccca
ggccctaaagatggcagaaggtaagaagctaaatgtttatactgatagccgttatgcttttgctactgcccatatccatggagaaatata
cagaaggcgtgggtggctcacatcagaaggcaaagagatcaaaaataaagacgagatcttggccctactaaaagccctctttctgc
ccaaaagacttagcataatccattgtccaggacatcaaaagggacacagcgccgaggctagaggcaaccggatggctgaccaag
cggcccgaaaggcagccatcacagagactccagacacctctaccctcctcatagaaaattcatcaccctctggcggctcaaaaaga
accgccgacggcagcgaattcgagcccaagaagaagaggaaagtctaaccggtcatcatcaccatcaccattgagtttaaacccg
ctgatcagcctcgactgtgccttctagttgccagccatctgttgtttgcccctcccccgtgccttccttgaccctggaaggtgccactcccac
tgtcctttcctaataaaatgagaaaattgcatcgcattgtctgagtaggtgtcattctattctggggggtggggtggggcaggacagcaa
gggggaggattgggaagacaatagcaggcatgctggggatgcggtgggctctatggcttctgaggcggaaagaaccagctgggg
ctcgataccgtcgacctctagctagagcttggcgtaatcatggtcatagctgtttcctgtgtgaaattgttatccgctcacaattccacacaa
catacgagccggaagcataaagtgtaaagcctagggtgcctaatgagtgagctaactcacattaattgcgttgcgctcactgcccgctt
tccagtcgggaaacctgtcgtgccagctgcattaatgaatcggccaacgcgcggggagaggcggtttgcgtattgggcgctcttccgc
ttcctcgctcactgactcgctgcgctcggtcgttcggctgcggcgagcggtatcagctcactcaaaggcggtaatacggttatccacag
aatcaggggataacgcaggaaagaacatgtgagcaaaaggccagcaaaaggccaggaaccgtaaaaaggccgcgttgctggc
gtttttccataggctccgcccccctgacgagcatcacaaaaatcgacgctcaagtcagaggtggcgaaacccgacaggactataaa
gataccaggcgtttccccctggaagctccctcgtgcgctctcctgttccgaccctgccgcttaccggatacctgtccgcctttctcccttcg
ggaagcgtggcgctttctcatagctcacgctgtaggtatctcagttcggtgtaggtcgttcgctccaagctgggctgtgtgcacgaacccc
ccgttcagcccgaccgctgcgccttatccggtaactatcgtcttgagtccaacccggtaagacacgacttatcgccactggcagcagc
cactggtaacaggattagcagagcgaggtatgtaggcggtgctacagagttcttgaagtggtggcctaactacggctacactagaag
aacagtatttggtatctgcgctctgctgaagccagttaccttcggaaaaagagttggtagctcttgatccggcaaacaaaccaccgctg
gtagcggtggtttttttgtttgcaagcagcagattacgcgcagaaaaaaaggatctcaagaagatcctttgatcttttctacggggtctgac
actcagtggaacgaaaactcacgttaagggattttggtcatgagattatcaaaaaggatcttcacctagatccttttaaattaaaaatga
agttttaaatcaatctaaagtatatatgagtaaacttggtctgacagttaccaatgcttaatcagtgaggcacctatctcagcgatctgtcta
tttcgttcatccatagttgcctgactccccgtcgtgtagataactacgatacgggagggcttaccatctggccccagtgctgcaatgatac
cgcgagacccacgctcaccggctccagatttatcagcaataaaccagccagccggaagggccgagcgcagaagtggtcctgcaa
ctttatccgcctccatccagtctattaattgttgccgggaagctagagtaagtagttcgccagttaatagtttgcgcaacgttgttgccattgc
tacaggcatcgtggtgtcacgctcgtcgtttggtatggcttcattcagctccggttcccaacgatcaaggcgagttacatgatcccccatgt
tgtgcaaaaaagcggttagctccttcggtcctccgatcgttgtcagaagtaagttggccgcagtgttatcactcatggttatggcagcact

gcataattctcttactgtcatgccatccgtaagatgcttttctgtgactggtgagtactcaaccaagtcattctgagaatagtgtatgcggcg
accgagttgctcttgcccggcgtcaatacgggataataccgcgccacatagcagaactttaaaagtgctcatcattggaaaacgttctt
cggggcgaaaactctcaaggatcttaccgctgttgagatccagttcgatgtaacccactcgtgcacccaactgatcttcagcatcttttac
tttcaccagcgtttctgggtgagcaaaaacaggaaggcaaaatgccgcaaaaaagggaataagggcgacacggaaatgttgaat
actcatactcttcctttttcaatattattgaagcatttatcagggttattgtctcatgagcggatacatatttgaatgtatttagaaaaataaaca
aataggggttccgcgcacatttccccgaaaagtgccacctgacgtcgacggatcgggagatcgatctcccgatcccctagggtctact
ctcagtacaatctgctctgatgccgcatagttaagccagtatctgctccctgcttgtgtgttggaggtcgctgagtagtgcgcgagcaaaa
tttaagctacaacaaggcaaggcttgaccgacaattgcatgaagaatctgcttagggttaggcgttttgcgctgcttcgcgatgtacggg
ccagatatacgcgtt

Supplemental Table 3. Normalized Gt kinetic constants.

Variant	k x 10^{-3} (s^{-1}) (normalized)	error (95% confidence interval)
**WT**	1.00	0.04
**E150K**	1.24	0.04
**E150R**	1.03	0.05
**E150G**	1.34	0.04
**E150K, N151S**	1.36	0.04
**E150K, N151D**	1.21	0.03
**N151D**	1.08	0.03
**E150G/N151D**	1.19	0.03
**E150G/N151G**	1.17	0.04
**N151S**	0.99	0.04

Gt activation assay of rhodopsin WT and nine mRho mutants. The results represent the kinetic rates of Gt activation, normalized to that of WT. The data were analyzed in the same way as described for Fig. 2D, for an independent experiment. The error represents the 95% confidence interval.

Supplemental Table 4. CIRCLE-seq nominated sites.

Chromosome	Start	End	Genomic Coordinate	Nuclease Read Count	Strand	Control Read Count	Site Sequence	Site Substitution Number	Site Seq. Gaps Allowed
chr3	30159808	30159831	chr3:30159808-30159831	1556	+	0	CATGGAGAATCACCCTATCAAGG	4	
chr13	67568213	67568235	chr13:67568213-67568235	1466	+	0			AGGGAAGATACAC-CTATCAGGG
chr7	42019204	42019227	chr7:42019204-42019227	1432	+	0	TAGGAAGAAAAACACTATCAAGG	5	
chr8	79093547	79093569	chr8:79093547-79093569	1390	-	0			TGGGCAGAATC-CACTATCATGG
chr7	41945544	41945567	chr7:41945544-41945567	1120	+	0	TAGGAAGAACAACACTATCAAGG	5	
chr10	74357979	74358002	chr10:74357979-74358002	1030	+	0	GTGGGAGAATAACACTATCTGGG	6	
chr12	9924894	9924917	chr12:9924894-9924917	1011	+	0	AGAGGAGAATCACACTATCATGG	4	

chr9	104933045	104933067	chr9:104933045-104933067	904	-	0			CAGGGAGAATCATG-TATCAGGG
chr1	38501105	38501128	chr1:38501105-38501128	782	-	0	TGGGAAGAACCACACCATGGTGG	6	
chr1	170868470	170868492	chr1:170868470-170868492	777	+	0			AGAGAAGAATCATGC-ATCACGG
chr6	23850425	23850448	chr6:23850425-23850448	670	+	0	AGCTAAGAATCACTCTATCTGGG	5	
chr6	115933938	115933961	chr6:115933938-115933961	656	+	0	CGGGGAGAATCACGCTATCATGG	1	
chr14	117785299	117785322	chr14:117785299-117785322	548	+	0	AGGAAAGAATCATACTATCACAG	5	
chr14	23893123	23893145	chr14:23893123-23893145	478	+	0			CTGAAAGAACCA-GCTATCATGG
chr17	10797169	10797192	chr17:10797169-10797192	456	+	0	AGGGAAGAAACACGCTACCAAGG	3	

chr18	55794796	55794819	chr18:55794796-55794819	360	-	0	CATAAAGAATCATACTATCAAAG	6	
chr11	97521486	97521509	chr11:97521486-97521509	358	+	0	TGGGAAGAATCACACAAGGAAGG	5	
chr9	67278966	67278989	chr9:67278966-67278989	336	+	0	AGGGAAGAGTTACGCTATGGGTG	6	
chr11	49151776	49151798	chr11:49151776-49151798	292	+	0			GGAGAAGAATCA-GATATCATGG
chr2	120880639	120880662	chr2:120880639-120880662	288	+	0	AGGTAAGAAACACGCTATCACTG	4	
chr13	46726662	46726684	chr13:46726662-46726684	274	+	0			AGGGAAGAACCACGC-ATCAGAG
chr15	13374875	13374898	chr15:13374875-13374898	268	+	0	ATGGAAAGATCACCCTATCTAGG	6	
chr2	180978998	180979021	chr2:180978998-180979021	266	-	0	ATGGAAGAATCACGCTATGAAGG	3	
chr2	80307721	80307743	chr2:80307721-80307743	244	-	0			TGGGAAAATTCAC-

									CTATCAGGG
chr12	111163279	111163302	chr12:111163279-111163302	210	-	0	ACAGAAGAGTCACACTATCAAAG	6	
chr5	151206505	151206528	chr5:151206505-151206528	210	-	0	AGAGATAAATCACACTATCTGGG	6	
chr11	102936674	102936696	chr11:102936674-102936696	178	-	0			AAGGAAGAA-CACCCTATCAGGG
chr7	133987375	133987398	chr7:133987375-133987398	172	+	0	AGAGAAGAATCATGCTACAAGGA	6	AGAGAAGAATCATGCTA-CAAGG
chr7	18797978	18798001	chr7:18797978-18798001	140	+	0	CTTGGAGAATCACCCTGTCATGG	5	
chr7	100364503	100364526	chr7:100364503-100364526	134	+	0	CATGAAAAATCACACGATCAAGG	5	
chr5	142103427	142103450	chr5:142103427-142103450	122	+	0	CCTGAAGAATAACACCATCAAGG	5	
chr8	56147935	56147958	chr8:56147935-56147958	118	-	0	GTTGGAGAATCCTGCTATCAAGG	6	

chr9	16171448	16171470	chr9:16171448-16171470	118	+	0			AGGGAAGAATCA-GCTCTCATGG
chr4	28762863	28762886	chr4:28762863-28762886	116	+	0	AAGGAAGCATCATGCTATCCAAG	6	
chrX	12297078	12297101	chrX:12297078-12297101	116	+	0	AGGGAGCAATCACGCTATCTTAG	5	
chr4	109720368	109720391	chr4:109720368-109720391	114	+	0	CTTGAAGAATCAGGCTATTAGGG	4	
chr9	61996822	61996845	chr9:61996822-61996845	112	+	0	TGGGAAGCACCACGCTATCAAGA	4	
chrX	45874430	45874453	chrX:45874430-45874453	108	+	0	CAAGGAAAATCACTCTATCATGG	5	AAGGAA-AATCACTCTATCATGG
chr5	46884253	46884276	chr5:46884253-46884276	102	-	0	TTAAAAGAATCACTCTATCAGAG	6	
chr14	83583642	83583665	chr14:83583642-83583665	96	-	0	TAGGAAGAACAACACTATCAAAG	6	
chr13	84802770	84802793	chr13:84802770-84802793	88	+	0	TTGGAAGAATCACA	6	

							CTCACAG CG		
chr5	93099575	93099597	chr5:93099575-93099597	74	-	0			TGGGAGGAAGC-CGCTATCAGGG
chr2	31951699	31951721	chr2:31951699-31951721	72	-	0			CGGGAAGAGACAC-CTATCTGGG
chr7	82522755	82522778	chr7:82522755-82522778	70	-	0	CAATAAGAACCACACTATCTGGG	6	
chr13	80400497	80400520	chr13:80400497-80400520	62	+	0	TAGGAAGAACCATGCTATGAAGG	5	
chr8	57718120	57718143	chr8:57718120-57718143	62	-	0	TGGGAAGACTCATCCTATCATGG	4	
chr2	95950095	95950118	chr2:95950095-95950118	60	-	0	ACAGAAGAATCATGCTATAAGGA	6	
chr1	73850588	73850611	chr1:73850588-73850611	54	-	0	TTAAGAGAATCAGGCTATCAAGG	6	
chrX	150590482	150590505	chrX:150590482-150590505	54	-	0	CTGGGAGGATCACACAATCACGG	5	

chr3	135415268	135415291	chr3:135415268-135415291	52	+	0	CCAGAAGAAGCATACTATCAAGG	5	
chr1	100911711	100911734	chr1:100911711-100911734	50	-	0	CTTGTAGAATCACACCATCATGG	5	
chr1	88617277	88617300	chr1:88617277-88617300	48	-	0	TGGGAAGATCCACGCTACTGGGG	6	
chr2	129815909	129815932	chr2:129815909-129815932	48	+	0	GGAATAGAATCATGCTATCAGAG	6	
chr3	30261981	30262003	chr3:30261981-30262003	46	+	0			CGGGAA-AGTCACTCTATCTAGG
chr6	54473703	54473726	chr6:54473703-54473726	46	+	0	GAGAAAGAAACACGCTACCAGAG	6	
chr14	78355477	78355500	chr14:78355477-78355500	44	-	0	TTGGAGGAAACACACTATTAGGG	6	
chr1	154420004	154420026	chr1:154420004-154420026	44	+	0			GGGGAAGAATCACAC-ATCATGA
chr16	20179693	20179716	chr16:20179693-20179716	42	+	0	CTGGAAGGATCACG	6	

							CTCTGGG<br>TG		
chr11	7025<br>3633	7025<br>3656	chr11:7025<br>3633-<br>70253656	40	+	0	AGGGGA<br>GAATCAC<br>GCTAATT<br>AAG	6	AGGGG<br>AGAATC<br>ACGCTA<br>ATTAAG<br>G
chr17	8894<br>2925	8894<br>2948	chr17:8894<br>2925-<br>88942948	40	-	0	CTGGTAG<br>AATCACC<br>CTATCAG<br>GA	4	
chr17	5112<br>2361	5112<br>2384	chr17:5112<br>2361-<br>51122384	38	+	0	GTAGAAG<br>AATCTCA<br>CTATCAA<br>AG	6	
chr5	6495<br>2566	6495<br>2589	chr5:64952<br>566-<br>64952589	38	+	0	GAGAAAG<br>AAGAATG<br>CTATCAT<br>GG	6	
chr6	7369<br>4906	7369<br>4929	chr6:73694<br>906-<br>73694929	34	+	0	TGTGAAG<br>AATCTTA<br>CTATCAG<br>GG	5	
chr14	8676<br>2669	8676<br>2692	chr14:8676<br>2669-<br>86762692	32	-	0	GGGGAG<br>GAAACAC<br>GCTATGA<br>AGG	4	
chr2	1644<br>9448<br>6	1644<br>9450<br>9	chr2:16449<br>4486-<br>164494509	32	+	0	AGGGAAG<br>AATTGCG<br>CTATAGG<br>GG	5	AGGGA<br>AGAATT<br>GCGCTA<br>T-AGGG
chr4	1004<br>8974<br>8	1004<br>8977<br>0	chr4:10048<br>9748-<br>100489770	32	-	0			CAGGAA<br>-<br>AATCAC<br>TCTATC<br>TTGG

chr7	136507152	136507175	chr7:136507152-136507175	32	-	0	AGGGAAGAAACCCACTATCAGGA	5	
chr9	93226682	93226705	chr9:93226682-93226705	32	+	0	TGGAAAGAATTACGCTAAAAGGG	5	
chr1	157452209	157452232	chr1:157452209-157452232	30	-	0	CCTTAAGAATCACACCATCTGGG	6	
chr3	26202366	26202389	chr3:26202366-26202389	30	+	0	AGGAAAGAATAATGCTATCAGGA	5	
chr11	107300021	107300044	chr11:107300021-107300044	28	+	0	AGGGGGGAAGCACGCTATCAAGG	4	
chr4	110846806	110846829	chr4:110846806-110846829	28	-	0	CAGGAAGAAAGACACAATCAAGG	5	
chr6	80314701	80314724	chr6:80314701-80314724	28	-	0	AGAGAAGAACCACACTAACTGGG	6	
chr12	12615833	12615856	chr12:12615833-12615856	26	-	0	GGAGAAGTATCACATTATCATGG	5	
chr14	121195095	121195118	chr14:121195095-121195118	26	-	0	AGGGAAGAAGAACATTATCAGGG	5	

chrX	60004767	60004789	chrX:60004767-60004789	26	-	0			TGGGAAGACTCA-GCTAACAGGG
chr10	60685756	60685779	chr10:60685756-60685779	24	-	0	GGAGAAGAATCACGCTGGGAGTG	6	
chr2	180493346	180493369	chr2:180493346-180493369	24	-	0	GTAGAAGAATCAGGCCATCAAGG	5	
chr3	158869936	158869959	chr3:158869936-158869959	24	+	0	TGACTAGAATCACTCTATCTGGG	6	
chr6	54206699	54206721	chr6:54206699-54206721	24	+	0			GAGGAAGAA-CACGCAATCAAGG
chr7	79769404	79769427	chr7:79769404-79769427	24	+	0	TGGGCAGAATCACTTTATCAAGG	4	
chr7	83734891	83734914	chr7:83734891-83734914	24	+	0	AGGGAGAAATCACGCTATGAGGC	5	
chr3	66466931	66466954	chr3:66466931-66466954	22	-	0	AAGGGTGAATCACGCTGACAAGG	6	
chr6	133525160	133525183	chr6:133525160-133525183	22	+	0	AAGGAAAAGTCACG	5	

							CTATCAA GA		
chr19	44462945	44462968	chr19:44462945-44462968	20	-	0	AGGGAGGAACCACGTTATCTGGC	6	
chr1	80397879	80397902	chr1:80397879-80397902	20	-	0	CAGGAAGCATCATGCTATCAAGC	4	
chr8	9810990	9811013	chr8:9810990-9811013	20	-	0	GAGAAAGAACCACACTATTATGG	6	
chr13	99926916	99926939	chr13:99926916-99926939	18	+	0	GTGGAGAAGTCAAGCTATCAGGG	6	
chr14	30627720	30627742	chr14:30627720-30627742	18	-	0			TGGGAAGAATC-CACTGTCATGG
chr14	47087281	47087304	chr14:47087281-47087304	18	+	0	AGCAATGAACCACGCCATCATGG	6	
chr19	53501417	53501440	chr19:53501417-53501440	18	-	0	AGGGGAGAAACACACCATCTAGG	6	
chr5	60531339	60531362	chr5:60531339-60531362	18	-	0	CTTGGAGAAGCACTCTATCACGG	5	
chr8	87333357	87333379	chr8:87333357-87333379	18	+	0			CTGGGAGA-TCACGC

									TGTCAG GG
chr9	5696 4889	5696 4912	chr9:56964 889- 56964912	18	-	0	CGGGAAG AATCAGG CTCAGAG AG	5	
chr11	7147 4583	7147 4606	chr11:7147 4583- 71474606	16	-	0	AATGAGA AATCACA CTATCAT GG	6	
chr15	7523 161	7523 184	chr15:7523 161- 7523184	16	-	0	CAGGAAG GTTCACA CTACCTA GG	6	
chr2	6247 1331	6247 1354	chr2:62471 331- 62471354	16	-	0	CAGGGGA AATGACA CTATCAA GG	6	
chr7	8132 3907	8132 3930	chr7:81323 907- 81323930	16	-	0	CCTGAAG AATCACA TTATCTG AG	6	
chr8	1144 1696 4	1144 1698 7	chr8:11441 6964- 114416987	16	+	0	CCAGAAG GATCACG ATATCAA GG	4	
chr10	7631 8783	7631 8806	chr10:7631 8783- 76318806	14	+	0	AGGGAAG AAGCACA CTACCAA AG	5	
chr18	4585 9291	4585 9314	chr18:4585 9291- 45859314	14	-	0	GAGGCAG AATCATG CCATCAA GG	5	
chr19	2292 2209	2292 2232	chr19:2292 2209- 22922232	14	+	0	TGTGAAG AACCACT	5	

							ATATCAGGG		
chr2	164518981	164519004	chr2:164518981-164519004	14	+	0	AGTCCAGAATCAAGCTATCCTGG	6	
chr2	27465262	27465284	chr2:27465262-27465284	14	+	0			TGGGAAGGA-CACGCTGTCAGGG
chr4	143749972	143749995	chr4:143749972-143749995	14	-	0	ATGGAAGAATCAGGGTATCAGGG	4	
chr6	135207211	135207233	chr6:135207211-135207233	14	+	0			CAGGAACAATCACGCC-TCACGG
chr6	148498089	148498112	chr6:148498089-148498112	14	+	0	ATGGAAGAAATATGCTATCATTG	6	
chr10	92441938	92441961	chr10:92441938-92441961	12	-	0	CAAGAAGACTCACACTATCGTGG	5	
chr10	92509244	92509267	chr10:92509244-92509267	12	+	0	CAGGAAGACCCACACTATCTGGA	6	
chr12	38357837	38357860	chr12:38357837-38357860	12	-	0	GTGGAAGATACATGCTATCAGAG	6	

chr16	4076254	4076276	chr16:4076254-4076276	12	-	0			TGGGAAGTATCA-GCTATCAAGA
chr1	35289108	35289131	chr1:35289108-35289131	12	+	0	CTATAAGAATCTTGCTATCATGG	5	
chr2	37048300	37048323	chr2:37048300-37048323	12	+	0	AGGGAAGCATAACACTATTAGGG	5	
chr5	119722389	119722412	chr5:119722389-119722412	12	+	0	TTTGAGGAAGCACTCTATCATGG	6	
chr5	52752734	52752757	chr5:52752734-52752757	12	+	0	TGAGAAAAATCATACTATCATGA	6	
chr8	3989349	3989371	chr8:3989349-3989371	12	+	0			CAGGAAGAATTTC-CTATCAAGG
chr8	5977643	5977666	chr8:5977643-5977666	12	+	0	AGGGGAGAAGTACACTATCAGAG	6	
chr9	31216419	31216441	chr9:31216419-31216441	12	+	0			AGGGAAGAA-CACGCAAACATGG

chr9	73494830	73494853	chr9:73494830-73494853	12	+	0	AAGGGAGAAGAACGCAATCATGG	6	
chrX	132371087	132371110	chrX:132371087-132371110	12	+	0	TGAGAAGAATCATGCCATCAAAG	5	
chr10	49014977	49015000	chr10:49014977-49015000	10	+	0	GAGGGAGAAGAAAGCTATCATGG	6	
chr12	81672706	81672729	chr12:81672706-81672729	10	-	0	CTGGAAGAATCCCAGGATCATGG	5	
chr15	52975121	52975144	chr15:52975121-52975144	10	+	0	AGGGAAGAATTACAGTATCAAGG	4	
chr15	71280724	71280746	chr15:71280724-71280746	10	-	0			TGGGAAGACTCACCCTA-CATGG
chr15	99791219	99791242	chr15:99791219-99791242	10	+	0	ATTGAGGAATAATGCTATCATGG	6	
chr16	74538607	74538630	chr16:74538607-74538630	10	+	0	ACTGAAGAAGCACCCTATCCCGG	6	
chr19	20611857	20611880	chr19:20611857-20611880	10	-	0	TGAGGTGAATCATGCTATCACGA	6	

chr1	144843650	144843673	chr1:144843650-144843673	10	-	nan	TTGAAAGAATCAACCAATCAAGG	6	
chr1	65877444	65877466	chr1:65877444-65877466	10	-	0			CAGGAAGAATTTC-CTATCAAGG
chr3	68838894	68838917	chr3:68838894-68838917	10	+	0	TTGGTGGAATCACACTACCATGG	6	
chr3	78906922	78906945	chr3:78906922-78906945	10	-	0	GAGGAAGAAACACGCTCACATGG	5	
chr6	112100615	112100638	chr6:112100615-112100638	10	+	0	TGGGAAGACACAAGCTATCATGG	4	
chr6	20582820	20582843	chr6:20582820-20582843	10	+	0	GGGGAAGATTCAAGCAATCATGG	4	
chr7	121678052	121678075	chr7:121678052-121678075	10	+	0	GAGGAAGAAGTACCCTATCACAG	6	
chr7	76173134	76173157	chr7:76173134-76173157	10	-	0	CAAGAAGAGCCACATTATCAAGG	6	
chrX	11879320	11879343	chrX:11879320-11879343	10	+	0	GTAGAGGAATCACTATATCAAGG	6	

chr10	10547293 9	10547296 2	chr10:105472939-105472962	8	+	0	AGGGAAAAATCACTTTATCATGA	5	
chr10	40685259	40685281	chr10:40685259-40685281	8	+	0			CGGGGAGAAGCA-GCTATCTTGG
chr10	94165778	94165801	chr10:94165778-94165801	8	+	0	AAGGAAGAAAAACGTTATCTGGG	6	
chr12	11820453 0	11820455 3	chr12:118204530-118204553	8	+	0	GTTTAAGAATCATTCTATCATGG	6	
chr12	80184734	80184757	chr12:80184734-80184757	8	+	0	CTGGGAGCTTCACTCTCTCAGGG	6	
chr13	92974342	92974365	chr13:92974342-92974365	8	+	0	AGGGAAGAAATGCATTATCAGGG	6	
chr14	37636877	37636900	chr14:37636877-37636900	8	-	0	GAGGGAGAAGAAAGCTATCATGG	6	
chr17	15203098	15203121	chr17:15203098-15203121	8	-	0	GGAGGAAAATCATGCTATCAAGA	6	
chr17	41977341	41977364	chr17:41977341-41977364	8	-	0	GTTGAAGAATCAAGCTATCAAGC	5	

chr1	103243839	103243862	chr1:103243839-103243862	8	+	nan	GTGGAAGAATCCAGCTAACCAGG	6	
chr2	162679943	162679966	chr2:162679943-162679966	8	+	0	CTGGGAGAACCACACAATCACAG	6	
chr2	63878131	63878154	chr2:63878131-63878154	8	+	0	ACAGCAGAATTACGCCATCACGG	6	
chr4	4911706	4911729	chr4:4911706-4911729	8	-	0	AGGGAACAATTATGCTATCAGGC	5	
chr5	86633984	86634007	chr5:86633984-86634007	8	-	0	AGGAAGGAAACACACAATCAGGG	6	
chr6	143978505	143978528	chr6:143978505-143978528	8	-	0	CTCTTTGTATCACGCTATCATGG	6	
chr7	38080658	38080681	chr7:38080658-38080681	8	+	0	GCTGAAGAATGACGCTAACAGGG	5	CTG-AAGAATGACGCTAACAGGG
chr7	61096347	61096370	chr7:61096347-61096370	8	+	0	TAGGAAGAACAACAATATCAAGG	6	
chr8	37938021	37938044	chr8:37938021-37938044	8	-	0	AAGGGAGAATTACCCTATCTTGG	6	

chr9	61855548	61855571	chr9:61855548-61855571	8	+	0	CGAGAAGAAACCAGCTATCAAGG	4	
chrX	158936023	158936045	chrX:158936023-158936045	8	+	0			AGTGAAGAATCACCCT-TCATGG
chr13	8361420	8361443	chr13:8361420-8361443	6	-	0	GGGTAAGAATTACACTATCTCTG	6	
chr14	66079995	66080018	chr14:66079995-66080018	6	-	0	AGAGAAAAATCACGTTATCAAGA	5	
chr15	43033972	43033995	chr15:43033972-43033995	6	+	0	CAGAAAGAATAATGCTATCAGGA	5	
chr15	90341570	90341593	chr15:90341570-90341593	6	+	0	TAATAGTAATCACGCTATCATGG	6	
chr17	19025570	19025593	chr17:19025570-19025593	6	-	0	AGGGAACAATTATGCTATCAGGC	5	
chr17	22448151	22448174	chr17:22448151-22448174	6	-	0	CTGGAAGAATCACAGGGTCAAGA	6	
chr18	30414215	30414237	chr18:30414215-30414237	6	-	0			GGGTATGAATCAC-CTATCAAGG

chr18	36723219	36723243	chr18:36723219-36723243	6	-	0			AGGGAAGAATCAACTCTATCTTGG
chr18	82649394	82649416	chr18:82649394-82649416	6	+	0			CGTGAAGAAACACGC-ATCAAGG
chr19	13457289	13457312	chr19:13457289-13457312	6	+	0	CTGGAAGAATCATTCTATCCATG	5	CTGGAAGAATCATTCTATCCATGG
chr19	17766508	17766531	chr19:17766508-17766531	6	-	0	AAGGGAGAATCAAGCTATAAGGA	6	
chr19	36338025	36338048	chr19:36338025-36338048	6	+	0	TGGGAAGAAACAAGCCACCATGG	5	
chr19	59551435	59551458	chr19:59551435-59551458	6	+	0	AAGGAAGAACCACCCTGACATGG	6	
chr1	192422166	192422189	chr1:192422166-192422189	6	-	0	AGGGAGGAACCACGCTAGCAGGT	5	
chr1	82924344	82924367	chr1:82924344-82924367	6	+	0	CAGAAAGAATCATGCCTTCAGGG	5	
chr2	103881743	103881766	chr2:103881743-103881766	6	+	0	GGTGTAGAAACACA	6	

							CTATCACAG		
chr2	143892729	143892752	chr2:143892729-143892752	6	+	0	ATGAAAGAATCAACCAATCAGGG	6	
chr2	81600719	81600742	chr2:81600719-81600742	6	-	0	CAGTAGAAATCACATTATCAAGG	6	
chr3	108305060	108305083	chr3:108305060-108305083	6	-	0	GGGGAAGAAACACGATGTCAAGG	4	
chr3	138255896	138255919	chr3:138255896-138255919	6	+	0	AGGGAAGAATAACGCTAGTAGGG	4	
chr3	54262311	54262334	chr3:54262311-54262334	6	+	0	AGAGAAGAAGTACGCGATCAAGG	5	
chr3	63166898	63166921	chr3:63166898-63166921	6	+	0	CAGGAAGAATCAATCCAGCCAGG	6	
chr4	138192244	138192267	chr4:138192244-138192267	6	+	0	TGGGAAGAATTACCCAATCACAG	5	
chr4	71387152	71387175	chr4:71387152-71387175	6	+	0	TGGAATGAATTATACTATCAAGG	6	
chr5	115733442	115733464	chr5:115733442-115733464	6	+	0			AGGAAAGAATGA

									CGCTA-CAGGG
chr5	22152508	22152531	chr5:22152508-22152531	6	+	0	AGCGAAGAATCACACTACAGAGG	6	
chr7	109493739	109493762	chr7:109493739-109493762	6	+	0	TAGGAAGAATGATGCAATCTGGG	6	
chr7	15820321	15820344	chr7:15820321-15820344	6	-	0	CAGCAAGAAACACGTTATCAGGG	4	
chr7	39823094	39823117	chr7:39823094-39823117	6	+	0	GAGGAAGAATCACTATATCAGGC	5	
chr8	50548030	50548053	chr8:50548030-50548053	6	+	0	TTGTAAGAATCACGCTAACAGGT	5	
chrX	50498574	50498597	chrX:50498574-50498597	6	+	0	CATCATGAATCATGCTATCATGA	6	

Supplemental References

1. Suh S*, et al.* (2021) Restoration of visual function in adult mice with an inherited retinal disease via adenine base editing. *Nat Biomed Eng* 5(2):169-178.
2. Chelstowska S, Widjaja-Adhi MAK, Silvaroli JA, & Golczak M (2017) Impact of LCA-Associated E14L LRAT Mutation on Protein Stability and Retinoid Homeostasis. *Biochemistry* 56(34):4489-4499.
3. Tsai SQ*, et al.* (2017) CIRCLE-seq: a highly sensitive in vitro screen for genome-wide CRISPR-Cas9 nuclease off-targets. *Nat Methods* 14(6):607-614.
4. An M*, et al.* (2024) Engineered virus-like particles for transient delivery of prime editor ribonucleoprotein complexes in vivo. *Nat Biotechnol*.
5. Clement K*, et al.* (2019) CRISPResso2 provides accurate and rapid genome editing sequence analysis. *Nat Biotechnol* 37(3):224-226.
